# Ultrasound-responsive Bi_2_MoO_6_-MXene heterojunction as ferroptosis inducers for stimulating immunogenic cell death against ovarian cancer

**DOI:** 10.1186/s12951-024-02658-3

**Published:** 2024-07-11

**Authors:** Shuangshuang Cheng, Ting Zhou, Yue Luo, Jun Zhang, Kejun Dong, Qi Zhang, Wan Shu, Tangansu Zhang, Qian Zhang, Rui Shi, Yuwei Yao, Hongbo Wang

**Affiliations:** 1 Department of Obstetrics and Gynecology, Union Hospital, Tongji Medical College, Huazhong University of Science and Technology, Hubei, 430022 Wuhan China; 2 Clinical Research Center of Cancer Immunotherapy, Hubei, 430022 Wuhan China; 3 Biomedical Materials Engineering Research Center, Collaborative Innovation Center for Advanced Organic Chemical Materials Co-constructed by the Province and Ministry, Hubei Key Laboratory of Polymer Materials, Ministry-of-Education Key Laboratory for the Green Preparation and Application of Functional Materials, School of Materials Science and Engineering, Hubei University, Wuhan, 430062 China

**Keywords:** Ovarian cancer, BMO-MXene, Sonodynamic therapy, Ferroptosis, Immunogenic cell death

## Abstract

**Background:**

Ovarian cancer (OC) has the highest fatality rate among all gynecological malignancies, necessitating the exploration of novel, efficient, and low-toxicity therapeutic strategies. Ferroptosis is a type of programmed cell death induced by iron-dependent lipid peroxidation and can potentially activate antitumor immunity. Developing highly effective ferroptosis inducers may improve OC prognosis.

**Results:**

In this study, we developed an ultrasonically controllable two-dimensional (2D) piezoelectric nanoagonist (Bi_2_MoO_6_-MXene) to induce ferroptosis. A Schottky heterojunction between Bi_2_MoO_6_ (BMO) and MXene reduced the bandgap width by 0.44 eV, increased the carrier-separation efficiency, and decreased the recombination rate of electron–hole pairs under ultrasound stimulation. Therefore, the reactive oxygen species yield was enhanced. Under spatiotemporal ultrasound excitation, BMO-MXene effectively inhibited OC proliferation by more than 90%, induced lipid peroxidation, decreased mitochondrial-membrane potential, and inactivated the glutathione peroxidase and cystathionine transporter protein system, thereby causing ferroptosis in tumor cells. Ferroptosis in OC cells further activated immunogenic cell death, facilitating dendritic cell maturation and stimulating antitumor immunity.

**Conclusion:**

We have succeeded in developing a highly potent ferroptosis inducer (BMO-MXene), capable of inhibiting OC progression through the sonodynamic-ferroptosis-immunogenic cell death pathway.

**Graphical Abstract:**

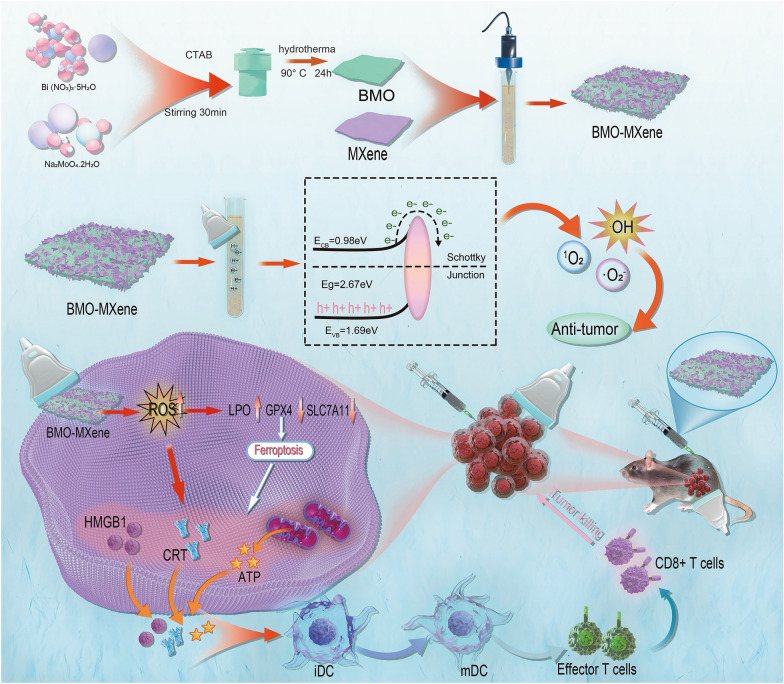

**Supplementary Information:**

The online version contains supplementary material available at 10.1186/s12951-024-02658-3.

## Introduction

Ovarian cancer (OC) stands as one of the most lethal gynecological malignancies, with a 5-year survival rate of 46% after diagnosis. This disease poses a major threat to the reproductive and overall health of women [1]. Unfortunately, owing to atypical symptoms that present during early-stage OC, most patients have an advanced-stage disease (International Federation of Gynecology and Obstetrics stages III–IV) at the time of diagnosis [2]. Currently, the standard treatment of epithelial OC comprises surgery followed by three–six cycles of carboplatin and paclitaxel chemotherapy. However, poor response rates, numerous adverse side effects, and drug resistance remain insurmountable challenges [3]. Furthermore, traditional treatments often do not meet the therapeutic expectations of patients seeking fertility preservation. In recent decades, immunotherapy has emerged as a promising avenue for cancer treatment, significantly improving outcomes in patients with cancer [4, 5]. However, immune-checkpoint blockade, which is currently the most commonly used immunotherapy, yields poor outcomes in patients with OC [6–8]. Therefore, developing novel, specific, safe, and effective therapeutic strategies for OC is critical.

Ferroptosis, an iron-dependent form of programmed cell death driven by excessive lipid peroxidation, primarily manifests as iron overload, lipid peroxidation, and reactive oxygen species (ROS) accumulation, which increases the sensitivity of cancer cells to chemotherapy, targeted therapy, and immunotherapy [9–12]. Several studies have revealed the potential of targeting ferroptosis in malignant tumors, including OC [12, 13]. Notably, ferroptosis triggers the release of tumor-associated antigenic burst, initiates tumor cell immunogenic cell death (ICD), and enhances antitumor immunity [14–18]. Therefore, a comprehensive therapeutic approach for overcoming multidrug resistance to chemotherapy and immunotherapy may involve inducing ferroptosis in tumor cells. However, the indiscriminate action of ferroptosis inducers throughout the body poses significant challenges, disrupting the homeostasis and physiological functions of normal cells, and causing serious toxic side effects [19–21]. For example, iron accumulation leads to ROS production, causing cell death, tissue damage, and the development of various kidney diseases, including acute kidney injury, fibrosis, renal cell carcinoma, and kidney transplant ischemia/reperfusion (I/R) injury [22]. Moreover, ferroptosis promotes interstitial fibrosis and inflammation in mouse models of chronic kidney disease, unilateral ureteral obstruction, and I/R. These detrimental therapeutic effects of ferroptosis inducers severely limit their clinical application. Therefore, there is an urgent need for developing specific tissue-responsive ferroptosis inducers. Notably, ferroptosis inhibitors ameliorated renal fibrosis by reducing MCP-1 secretion and macrophage chemotaxis [23].

In recent years, novel antitumor therapeutic modalities based on functional nanoparticles (NPs) have been extensively investigated, offering more precise, efficient, and noninvasive cancer treatments [24–31]. NPs can be effectively enriched in tumor tissues owing to their nanoscale effects and can be activated molecules under near-infrared light, ultrasound (US), or magnetic field excitation, disrupting tissue redox homeostasis and inhibiting tumor progression, without damaging normal tissues [32, 33]. Notably, US exhibits higher tissue penetration (> 10 cm in soft tissues) than near-infrared light, which is limited to the mm range. This characteristic renders sonodynamic therapy (SDT) an attractive strategy to effectively overcome the major limitations of photodynamic therapy and photothermal therapy in deep-tissue tumors [34]. In SDT, US not only activates the acoustic sensitizer, leading to cancer cell death, but also induces a secondary effect, initiating a series of cellular stress and immune reprogramming events. This multifaceted approach enhances the immunogenicity of tumor cells, improves the tumor immune microenvironment and inhibits tumor progression [35]. Although the feasibility of SDT as an antitumor-treatment modality has been consistently demonstrated [36–39], most acoustic sensitizers, including titanium dioxide, transition metal oxides, and noble metal NPs, exhibit low ROS yields due to excessively fast electron (e^−^) and hole (h^+^) complexation rates, which limits the efficacy of SDT [40]. Therefore, developing efficient inorganic acoustic sensitizers is crucial for the clinical applications of SDT.

Bismuth molybdate (Bi_2_MoO_6_, or BMO) is an important member of the Aurivillius family of layered compounds, recognized for its excellent photocatalytic properties and uniquely layered structure. BMO has an alternating [Bi_2_O_2_]^2+^ and [MoO_4_]^2−^ ion-layer structure, capable of generating strong spontaneous polarization and inherent built-in electric fields. This structure confers good electrical conductivity, visible-light absorption, and high stability. Notably, BMO exhibits good piezoelectric properties. During US vibration, BMO is polarized, creating a built-in electric field. The resulting piezoelectric potential serves as a potent driving force to promote charge separation and inhibit the complexation generated by US. Consequently, the separated e^–^ and h^+^ can migrate to opposite surfaces, where they undergo redox reactions, and produce ROS under US stimulation [41, 42]. BMO has a two-dimensional (2D) sheet-layer structure and a larger surface area than cubic piezoelectric NPs, which helps expand the mechanical energy-trapping region by exposing more redox-active sites and facilitating catalytic reactions. In addition, the 2D morphology facilitates the formation of a large contact area between the heterojunction and other 2D materials, enabling rapid transmission of electric charge [43].

However, clinical application of BMO is limited by its highly cytotoxic nature and the low ROS production rates due to the rapid complexation of electron–hole pairs. These limitations can be effectively addressed by constructing BMO-related heterojunctions. MXenes, a new family of 2D transition metals, are characterized by strong full-spectrum absorption, transition metal C with good hydrophilicity, excellent carrier mobility, and abundant functional groups owing to the presence of an H or O end groups [44–46]. MXenes possess submetallic properties and can bind to n-type semiconductors to form Schottky junctions [47]. At the semiconductor/metal interface, semiconductor photoelectrons are transferred from the semiconductor to MXene to balance the Fermi energy levels of both NPs, forming a Schottky barrier [48]. The existence of the Schottky barrier limits the recombination of e^−^ and h^+^ pairs.

**Fig. 1:**
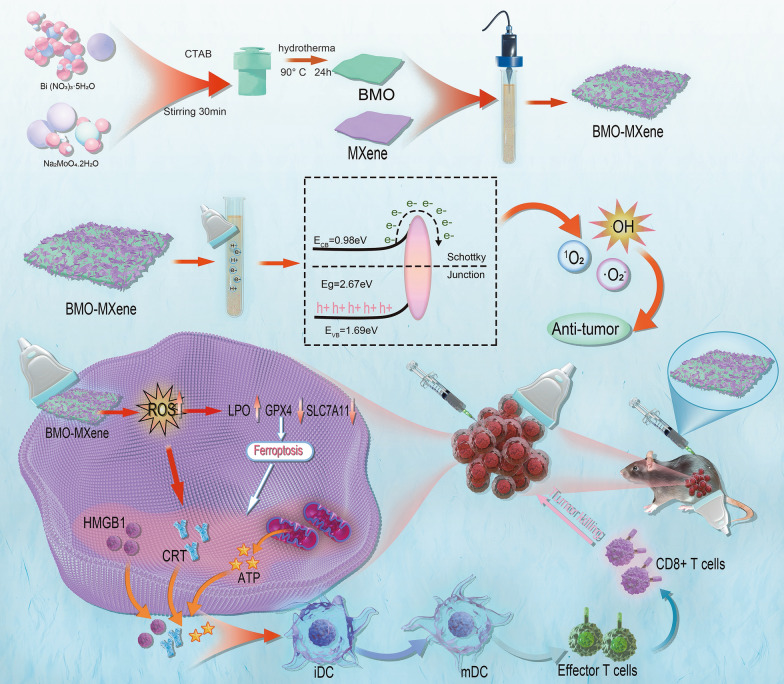
Schematic illustration of the antitumor mechanism of 2D piezoelectric BMO-MXene. Under excitation of ultrasound, sonosensitizer BMO-MXene acted as ferroptosis inducer stimulating immunogenic cell death and enhancing anti-tumor immunity in Ovarian Cancer

In this study, we developed SDT-based ferroptosis immunotherapy as a novel therapeutic modality for OC treatment. Leveraging the noninvasive and deep-penetrating nature of US, we constructed BMO-MXene, a 2D composite piezoelectric nanosheet with excellent acoustic-response capabilities. After cells phagocytose BMO-MXene, exogenous US applied at the tumor site drives BMO-MXene to initiate the catalyzed ROS production, enabling more tissue-controllable therapies. The acoustic response of BMO-MXene promoted tumor cell ferroptosis, induced ICD, and improved the immune microenvironment in OC tissues. In summary, we constructed a US-responsive nanosheet to increase ROS production through a Schottky heterojunction constructed with BMO-MXene, thereby enhancing ferroptosis and ICD and inhibiting OC (Fig. 1).

## Materials and methods

### Materials

Sodium molybdate dihydrate and bismuth nitrate pentahydrate were obtained from Sinopharm Chemical Reagent Co., Ltd. (Shanghai, China). MXene was purchased from Beike 2D Materials Co., Ltd. ID8 cells and SKOV3 cells were purchased from Yuchi Biotechnology Co., Ltd. (Shanghai, China). The CCK-8 Reagent (40203ES60), Calcein-AM/PI Reagent (40747ES76), Reactive Oxygen Species Assay Kit (50101ES01), and Annexin V-FITC/PI Apoptosis Detection Kit (40302ES20) were purchased from Shanghai Yeasen BioTechnologies co., Ltd. (Shanghai, China). C11-Bodipy 581/591 (D3861) was purchased from Thermo Fisher Scientific (Waltham, MA, USA). Lyso-Tracker Green (C1047S), Mitochondrial membrane potential assay kit with JC-1(C2006), ATP assay kit, Mouse IFN-γ ELISA Kit (High-sensitive) (PI507), and Mouse TNF-α ELISA Kit (PT512) were purchased from Beyotime Biotechnology (Jiangsu, China). DAPI (216276) and Tunel kit (11684817910) were obtained from Roche (Basel, Switzerland). The antibodies RIPK1 (17519-1-AP), MLKL (21066-1-AP), GSDMD (20770-1-AP), Bcl-2 (26593-1-AP), BAX (50599-2-Ig), GPX4 (67763-1-Ig), SLC7A11/xCT (26864-1-AP), CRT (10292-1-AP), Na^+^-K^+^-ATPase (14418-1-AP), and β-actin (66009-1-Ig) were obtained from Proteintech (Rosemount, IL, USA). The antibodies GSDME (ab215191), Ki-67 (ab16667), CD80 (ab134120), CD86 (ab119857), CD8 (ab217344), CD3 (ab135372), and IFN-γ (ab224297) were obtained from Abcam (Cambridge, UK). HMGB1 ELISA kit (RX101141H) was acquired from Quanzhou Ruixin Biotechnology Co., Ltd (Fujian, China). All chemicals and reagents used in this study were of analytical grade and used without any further purification.

### Synthesis of Bi_2_MoO_6_ (BMO) nanosheets

A mixture containing sodium molybdate dihydrate (1 mmol), Bismuth nitrate pentahydrate (2 mmol), hexadecyl trimethyl ammonium bromide (CTAB; 0.05 g) was dispersed in 80 ml deionized water and stirred for 30 min. The solution was then transferred to a reactor at 90° for 24 h. After the reaction, deionized water was used to wash the obtained powders until no foam is formed.

### Synthesis of 2D piezoelectric BMO-MXene nanosheets

The solution containing BMO (10 mg) and MXene (10 mg) was mixed with a cell commuter for 1 h and centrifuged immediately. The obtained powder was dried in an oven.

### Nanosheets characterization

A scanning electron microscope equipped with a field emission (SEM, JSM7100F, JEOL, JP), an electron microscope with transmission (TEM, JEM-2100F), and a high-resolution transmitted electron microscopy (HRTEM) were used to investigate the morphology of BMO, MXene, and BMO-MXene nanosheets. The crystalline phase of several nanosheets were determined by X-ray diffraction (D8A25, Bruker, Germany). X-ray photoelectron spectroscopy (XPS, ESCALAB 250Xi, Thermo Scientific, USA) was implemented to explore the elemental composition of several nanosheets. An electrochemical workstation (CHI660E, China) was carried out to analyze the sonocurrents of various nanosheets. To investigate the piezoelectric and ferroelectric properties, piezoresponse force microscopy (PFM, Bruker Dimension FastScan) was performed. Dynamic light scattering (DLS) data was obtained with Malvern Zetasizer Nano ZS90 used to characterize nanodiameters. UV–vis diffuse reflectance spectra data was acquired with Shimadzu UV-3600i Plus. PL spectra data was obtained from Edinburgh FLS 1000.

### ROS detection

3-diphenylisobenzofuran (DPBF), Nitroblue tetrazolium (NBT) in dimethylsulfoxide solution (2 μg/mL), and terephthalic acid solution (TA, 600 μg/mL) were employed as probes to identify singlet oxygen (^1^O_2_), superoxide (·O^−2^), and hydroxyl radicals (·OH) under US excitation respectively. In short, US was used to stimulate the mixture of BMO, MXene, or BMO-MXene and probe (DPBF, NBT, or TA) at intervals of 3 min till 15 min without light. Then the absorbance was measured at the corresponding time points. The DCFH-DA kit was implemented to measure intracellular ROS by using Fluorescence Microscope and a flow cytometer (Ex/Em: 480/525 nm). The Flowjo software was executed to process the acquired data.

### Cell culture

Yuchi Biotechnology Co., Ltd. (Shanghai, China) was the supplier of ID8 cells and SKOV3 cells. They were incubated in high-glucose DMEM or McCoy's 5A complete medium with 10% FBS and 1% penicillin–streptomycin in a 37 °C incubator with 5% CO_2_. Human bone marrow mesenchymal stem cells (hBMSC) were harvested from iliac crests of healthy donors from the Department of Orthopedics, Union Hospital, with signed informed consent. hBMSC were isolated by density gradient centrifugation. The DMEM/F12 complete media containing 10% Gibco FBS and 1% penicillin–streptomycin was used to cultivate the cells. In the experiments, second or third generation hBMSC were used gradually. For the following experiments, the solution of trypsin–EDTA (GIBCO, Invitrogen) was used to digest the cells for about 1 min.

### CCK8 assay to detect cytotoxicity of BMO-MXene

The hBMSC (per well containing 1.5 × 10^4^ cells) were cultivated on 48 well plates overnight, with three replicate wells set up for each group. After plastered, cells were transfected with BMO, MXene, and BMO-MXene at 50, 80, 100, and 200 μg/mL concentrations and incubated for further 8 h. The hBMSC without nanosheets treatment were denoted as control. The hBMSC lacking nanosheets cocultured served as the control. ID8 cells and SKOV3 cells (per well containing 1.5 × 10^4^ cells) were grown on 48 well dishes for 24 h. Following that, cells in every well were transfected by BMO, MXene, and BMO-MXene at concentration of 50, 80, and 100 µg/mL respectively, and incubated for additional 8 h. Then the cells were treated either with or without US (1.0 MHz, 1.5 W/cm^2^, 50% duty cycle, 3 min). The cell viability in each well was tested using a CCK8 kits.

### Calcein-AM/PI cell staining

Calcein-AM/PI was utilized to further determine cell viability. In brief, ID8 cells were treated with or without US (1.5 W/cm^2^, 1.0 MHz, 50% duty cycle, 3 min) after being transfected with BMO, MXene, and BMO-MXene (50 µg/mL) for 24 h. Then the cells were washed three times and treated for 20 min in the dark with 2 mol/L Calcein-AM and 4 mol/L propidium iodide (PI). Finally, fluorescence microscope (Olympus IX71, Tokyo, Japan) was deployed to measure the live cells in green and the dead cells in red. The cells that underwent no US or nanosheets treatment were designated as control.

### Apoptosis detection in vitro

The detrimental effect of nanosheets on ID8 tumor cells was evaluated by Annexin V-FITC/PI Apoptosis Detection Kit. Cell processing steps were the same as live and dead cell staining. The treated cells were digested with EDTA-free trypsin, then collected and washed twice with PBS at 4 °C. 5 μL of Annexin V-FITC and 10 μL of PI staining solution were used for labeling. Finally, the cells were collected and quantified using a flow cytometer (Becton Dickinson Company of America). To process the given data, the Flowjo program was utilized.

### Measurement of mitochondrial membrane potential

JC-1 Detection Kit was used to evaluate changes in mitochondrial membrane potential of different treatments on ID8 cells and SKOV3 cells. Cell processing steps were the same as live and dead cell staining. The treated cells were digested with EDTA-free trypsin, then collected and washed. Cells were washed with PBS and incubated with JC-1 working solution for 20 min. The probe that was not bound to the cells was then sufficiently removed. Inverted fluorescence microscopy was performed to observe the mitochondrial membrane potential of different groups of cells.

### Detection of lipid peroxidation level

The C11-BODIPY Kit was employed to determine Lipid peroxidation level of each treatment on OC. In brief, cells (per well containing 2 × 10^6^ cells) were planted into a plate with six wells and incubated for 24 h. After transfected by BMO, MXene, and BMO-MXene (50 µg/mL) for 24 h, ID8 cells were treated with or without US (1.5 W/cm^2^, 1.0 MHz, 50% duty cycle, 3 min), and cells not receiving US or nanosheets therapy were referred to as control. After being cleaned with PBS, the cells were incubated for an hour with the C11 BODIPY 581/591 working solution. The amount of lipid peroxidation was next evaluated using fluorescence microscopy.

### The performance of BMO-MXene in vivo

All animals in this study were 6-week-old, female C57BL/6 mice, purchased from Beijing Vital River Laboratory Animal Technology Co., Ltd. All animals were raised in the SPF environment of the Animal Experiment Center of Tongji Medical College of Huazhong University of Science and Technology, and the experimental operation conformed to the ethics and operation specifications formulated by the Animal Ethics Committee (IACUC Number:3356).

Phosphate buffer was used to resuspend ID8 cells to a density of 1 × 10^9^/mL. A transplant tumor model was constructed by subcutaneous injection of ID8 cell suspension 100ul into the right abdomen of the female C57BL/6 mice. After the tumor volume reached 100 mm^3^, the mice were divided into four groups: PBS, PSB + US, BMO-MXene, BMO-MXene + US. Each group's mice received intratumoral injections of BMO-MXene (2 mg/mL, 5 mg/kg) or PBS in the same volume. In the US groups, the irradiation (1.5 W/cm^2^, 1.0 MHz, 50% duty cycle, 15 min) was applied on 1d, 4d and 7d following injection. Every three days, the four groups of mice had their body weight and tumor breadth (a) and length (b) measured. Tumor volume was then determined using the formula V = (b × a^2^)/2. The experiment was stopped and the mice's subcutaneous tumors were divided once the tumors in the BMO-MXene + US group had greatly decreased. The tumor tissues were fixed with 4% paraformaldehyde and paraffin-embedded to produce tissue sections for subsequent Hematoxylin and eosin (H&E) staining, immunohistochemical (IHC) staining and immunofluorescence (IF) staining analysis. The morphological and structural characteristics of cells were observed by H&E staining. DNA breaks during apoptosis were measured by terminal deoxynucleotidyl transferase dUTP nick-end labeling (TUNEL). Ki-67 antibody IHC was performed to assess the proliferation index of tumor cells. Furthermore, GPX4 and SLC7A11 IHC staining were crucial markers to assess cell ferroptosis. To confirm the effects of BMO-MXene on the immunological microenvironment, IHC staining of HMGB1, Foxp3, CRT, and CD8 and IF staining of CD3^+^CD8^+^T cells, IFN-γ^+^CD8^+^ T cells and CD80^+^CD86^+^DC were done.

### Western blot

Cells were well lysed by a protease lysate containing RIPA. The protein was denatured adding 5 × loading buffer and boiling for 10 min. Equal amounts of total protein were separated by SDS-PAGE and transferred to PVDF membrane. Then, PVDF membrane was blocked with TBST containing 1% skim milk powder for 2 h. Place the blocked PVDF membrane in a primary antibody and incubate overnight in a 4 °C freezer. The secondary antibody was incubated at room temperature for 2 h. The luminescence imager takes the exposure and acquires the image.

### RNA isolation and quantitative real-time PCR (qRT-PCR)

Trizol reagent (Takara, Japan) was used to extracted total RNA from cells according to instructions for the kit. The Hiscript qRT SuperMix (Vazyme, Nanjing, China) was used to reverse-transcribe RNA to cDNA. Utilizing Universal SYBR Green Fast qPCR Mix from Abclonal in Wuhan, China, real-time PCR studies were carried out on the CFX Connect Real-Time PCR Detection System (Bio-Rad, Hercules, CA, USA). Using GAPDH as an internal benchmark. The 2^−ΔΔCT^ method was selected to calculated the qualified expression. The primers to be used were listed in Table 1.

**Table 1:** Genes and corresponding primer sequences used for RT-qPCR

Gene		Primer sequence
GPX4	Forward Reverse	5′-GGAGCCAGGGAGTAACGAAG-3′ 5′-CGGTGTCCAAACTTGGTGAAG-3
NOX1	Forward Reverse	5′-CGCTGCCATCGACTACATCA-3′ 5′-CCATTTACCCACACCACGGA-3′
ACSL4	Forward Reverse	5′-CGCTGCCATCGACTACATCA-3′ 5′-CCATTTACCCACACCACGGA-3′
PTGS2	Forward Reverse	5'-TGTACGGGGTTTGTGACTGG-3' 5'-TTGTGGGCTAGCACATAGGC-3'
β-actin	Forward Reverse	5′-CTCCATCCTGGCCTCGCTGT-3′ 5′-GCTGTCACCTTCACCGTTCC-3′

### Measurement of HMGB1, TNF-α and IFN-γ levels secreted by OC cells by ELISA

HMGB1, TNF-α and IFN-γ levels were detected by double antibody sandwich enzyme-linked immunosorbent assay (ELISA). The supernatant of treated cells or serum was extracted and centrifuged to remove the sediment. According to the protocol provided by the reagent supplier, the OD value was measured at 450 nm wavelength of the enzyme marker. The concentration of HMGB1, TNF-α and IFN-γ in the samples was calculated according to the fitted calibration curve.

### Intracellular ATP assay

The Beyotime ATP Assay Kit was used to measure intracellular ATP content. In brief, the treated cells were lysed with a specific solution, then centrifuged at 12000*g* for 5 min at 4 °C, and the supernatant was collected for subsequent measurement. After adding the appropriate amount of ATP assay working solution and mixing with a certain amount of cell supernatant, the RLU value or CPM was measured by a multifunctional enzyme marker with luminometer function, and then the concentration of ATP in the samples was calculated according to the plotted standard curve.

### Fluorescence co-localization detected by Lyso-Tracker Green

ID8 cells or SKOV3 cells were co-cultured with BMO-MXene containing RhB for 3 h or 6 h at 37 °C. After washing with PBS, cells were fixed with 4% paraformaldehyde for 30 min and fluorescence images were acquired using a confocal scanning microscope (LSM780).

### Biosafety of BMO-MXene in vivo

Mice blood and organs (heart, liver, spleen, lung, kidney) were collected before and after BMO-MXene + US treatment on days 7 and 14, respectively. Blood was used to analyze changes in routine blood and blood biochemical indicators. Organs were used for H&E staining analysis.

### Statistical analysis

All data were presented as mean ± standard deviation. The statistical analysis of the data over three groups was performed using one-way ANOVA with Dunnett’s post-hoc test to calculate the significant difference (*p < 0.05, **p < 0.01, and ***p < 0.001).

## Results and discussion

### Characterization of BMO-MXene

**Fig. 2:**
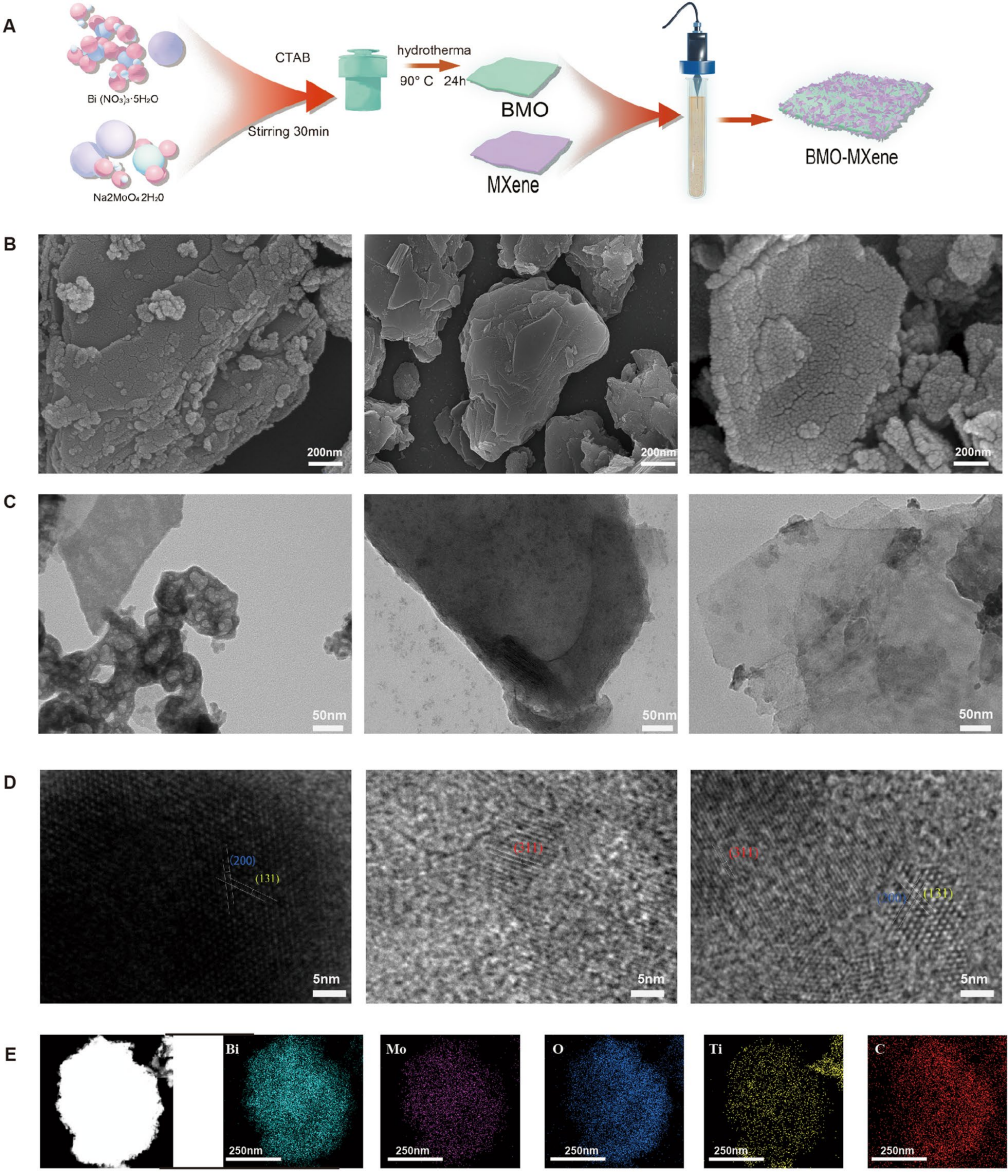
Characterization of BMO-MXene. **A** Synthesis process of BMO-MXene nanosheets. **B** SEM of BMO, MXene and BMO-MXene. **C** TEM of BMO, MXene and BMO-MXene. **D** HRTEM of BMO, MXene and BMO-MXene. **E** EDS of BMO-MXene

**Fig. 3:**
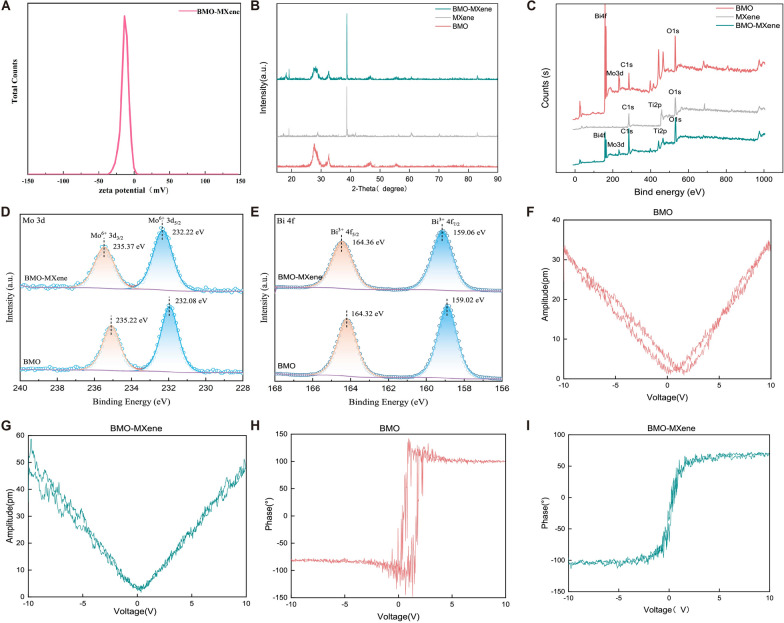
**A** Zeta potential of BMO-MXene. **B** XRD patterns of BMO, MXene, and BMO-MXene. **C** XPS spectra of BMO, MXene and BMO-MXene. **D** XPS Mo 3d spectrum of BMO and BMO-MXene. **E** XPS Bi 4f spectrum of BMO and BMO-MXene. **F, G** Amplitude curve of BMO and BMO-MXene obtained from PFM. **H, I** Phase curve of BMO and BMO-MXene obtained from PFM

BMO nanosheets were synthesized using a hydrothermal method. MXene and BMO were then mixed at a 1:1 ratio to form a BMO-MXene complex via electrostatic adsorption under ultrasonication (Fig. 2A). Both BMO and MXene exhibited irregular morphological sheet structures. Upon combination of BMO and MXene, the 2D structure was retained, albeit with a rougher crystal plane (Fig. 2B–D). The surface of BMO-MXene nanosheets, exhibited relatively uniform distributions of C, Ti, O, Mo, and Bi, with C and Ti originating from MXene and O, Mo, and Bi were from BMO (Fig. 2E). The Zeta potential of BMO-MXene was – 13.3mV (Fig. 3A). Atomic force microscopy (AFM) results confirmed that the size of BMO-MXene was in the range of 300–500 nm (Figure S1A). Dynamic light scattering (DLS) results of BMO-MXene showed a single peak, suggesting that BMO-MXene nano-size was more homogeneous and that the nanosystem was stable (Figure S1B).

X-ray diffraction patterns (Fig. 3B) revealed characteristic peaks of BMO at 28.31°, 32.53°, 46.73°, and 55.44°, corresponding to the (131), (200), (202), and (331) crystal planes of BMO, and matched the standard card of Bi_2_MoO_6_ (PDF#21-0102). The main characteristic diffraction peaks of MXene were located at 66.76°, 83.22°, 96.81°, and 111.84°, corresponding to the (311), (400), (420), and (422) surfaces of MXene, respectively, and were consistent with the standard card for single-layer MXene (PDF#02-0942). These findings were consistent with our high-resolution transmission electron microscopy results. X-ray photoelectron spectroscopy (XPS) revealed that BMO exhibited four main peaks corresponding to Bi4f, C1s, Mo3d and O1s. Three main peaks (C1s, O1s, and Ti2p) were detected for MXene, and five major peaks (Bi4f, C1s, Mo3d, O1s, and Ti2p) were detected for BMO-MXene, indicating successful complex formation between BMO and MXene (Fig. 3C). In the Mo3d spectrum, the two typical peaks at ~ 232.08 eV and ~ 235.22 eV were attributable to Mo^6+^3d_5/2_ and Mo^6+^3d_3/2_, respectively. Similarly, in the Bi4f spectrum, the two typical peaks at ~ 159.02 eV and ~ 164.32 eV were assigned to Bi^3+^4f_7/2_ and Bi^3+^4f_5/2_, respectively. In addition, both the Mo3d and Bi4f peaks exhibited slightly increased values after BMO complexation with MXene, confirming that BMO-MXene was successfully constructed [49] (Fig. 3D, E). The piezoelectric properties of BMO and BMO-MXene were measured via piezoelectric microscopy (PFM). A clear 180° phase shift in the phase hysteresis loop image and a typical butterfly loop in the amplitude curve consistently demonstrated the piezoelectric properties of BMO and BMO-MXene. Notably, the hysteresis loop of BMO-MXene was significantly narrower than that of BMO, indicating that small voltage changes could induce phase reversal (Fig. 3H, I). The butterfly ring structure of BMO-MXene suggests that smaller voltage changes corresponded to greater amplitude changes, compared to that of BMO as a reference (Fig. 3F, G). These PFM results demonstrate the enhanced piezoelectric performance of BMO-MXene over BMO.

### Acoustic dynamic performance of BMO-MXene

**Fig. 4:**
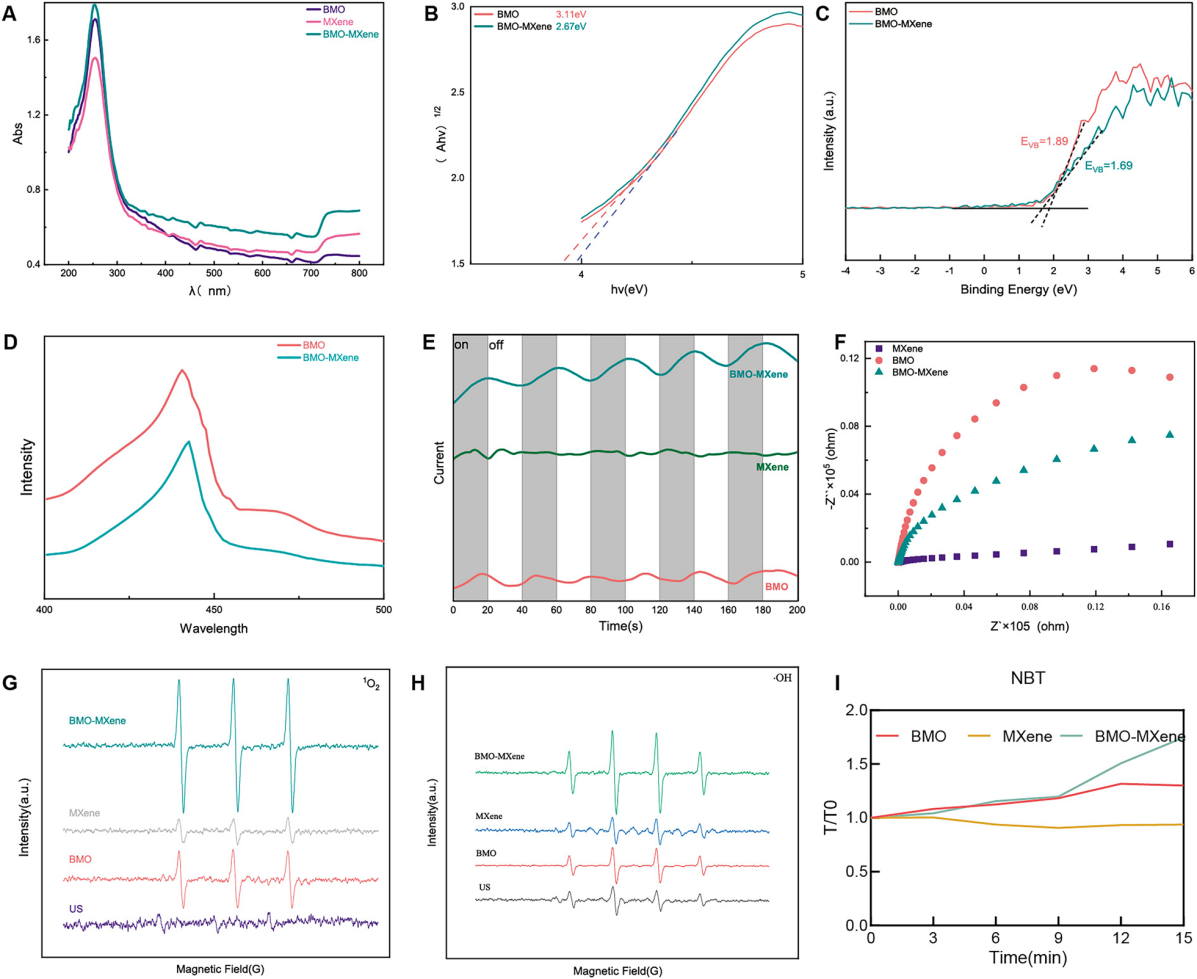
SDT performance of BMO-MXene. **A** UV–vis adsorption spectrum of BMO, MXene and BMO-MXene. **B** band gap energy of BMO and BMO-MXene. **C** Valence band of BMO and BMO-MXene. **D** Photoluminescence spectra of BMO and BMO-MXene.** E** Photocurrent density curves of BMO, MXene and BMO-MXene under US. **F** EIS of BMO, MXene and BMO-MXene under US. **G** ^1^O_2_ of BMO, MXene and BMO-MXene detected by ESR under US excitation.** H** ·OH of BMO, MXene and BMO-MXene detected by ESR under US excitation.** I** ·O^2−^ of BMO, MXene and BMO-MXene by NBT under US excitation

Ultraviolet–visible (UV–vis) diffuse reflectance spectroscopy data indicated that BMO-MXene exhibited a higher absorption spectrum than that of BMO alone, suggesting that complexation between BMO and MXene enhanced light absorption (Fig. 4A). The band gaps of BMO and BMO-MXene were estimated using Kubelka–Munk plots based on UV–vis absorption spectra. The band gap of BMO-MXene (2.67 eV) was lower than that of BMO (3.11 eV), indicating that less US energy was required for BMO-MXene activation (Fig. 4B). Moreover, analysis of the valence band spectra of the nanosheets was conducted using XPS data. The valence band energies of BMO and BMO-MXene were 1.89 eV and 1.69 eV, respectively, suggesting that BMO-MXene was more strongly reduced than BMO (Fig. 4C). Photoluminescence spectra were used to analyze the efficiency of electron–hole pair separation, revealing lower peaks for BMO-MXene than for BMO, suggesting that the BMO-MXene Schottky heterojunction formed by the composite effectively prevented the recombination of electron–hole pairs under US irradiation (Fig. 4D). An electrochemical workstation was used to study the electrochemical-impedance sound (EIS) and sonocurrent responses of different nanosheets; five repeated on/off cycles were performed under US irradiation (1.5 W/cm^2^, continuous at 1 MHz). The EIS of BMO-MXene was weaker than that of BMO, and the sonocurrent intensity of BMO-MXene was stronger than those of BMO and MXene (Fig. 4E, F). This result demonstrated that more charge transfer was induced at the heterointerface between BMO and MXene than from the individual materials. Examination of the the ability of nanosheets to produce different types of ROS was performed at different times following US stimulation. Electron spin resonance (ESR) revealed that BMO-MXene exhibited stronger singlet-oxygen excitation signals (Fig. 4G). DPBF degradation (based on ESR results at 420 nm) was observed to be the most rapid in the BMO-MXene group after 15 min of US stimulation (Figure S2), providing further evidence of the excellent singlet-oxygen production capability of BMO-MXene. Regarding ·OH, the BMO-MXene group demonstrated stronger absorption peaks after 15 min of US excitation (Figs. 4H, S3). Under SDT, the NBT characteristic peak at 525 nm in the BMO-MXene group increased sharply, indicating increased ·O^2−^ generation (Figs. 4I, S4). These results indicate the excellent ability of the BMO-MXene to produce ROS. Collectively, our results confirmed the superior SDT performance of BMO-MXene.

### In vitro antitumor performance

**Fig. 5:**
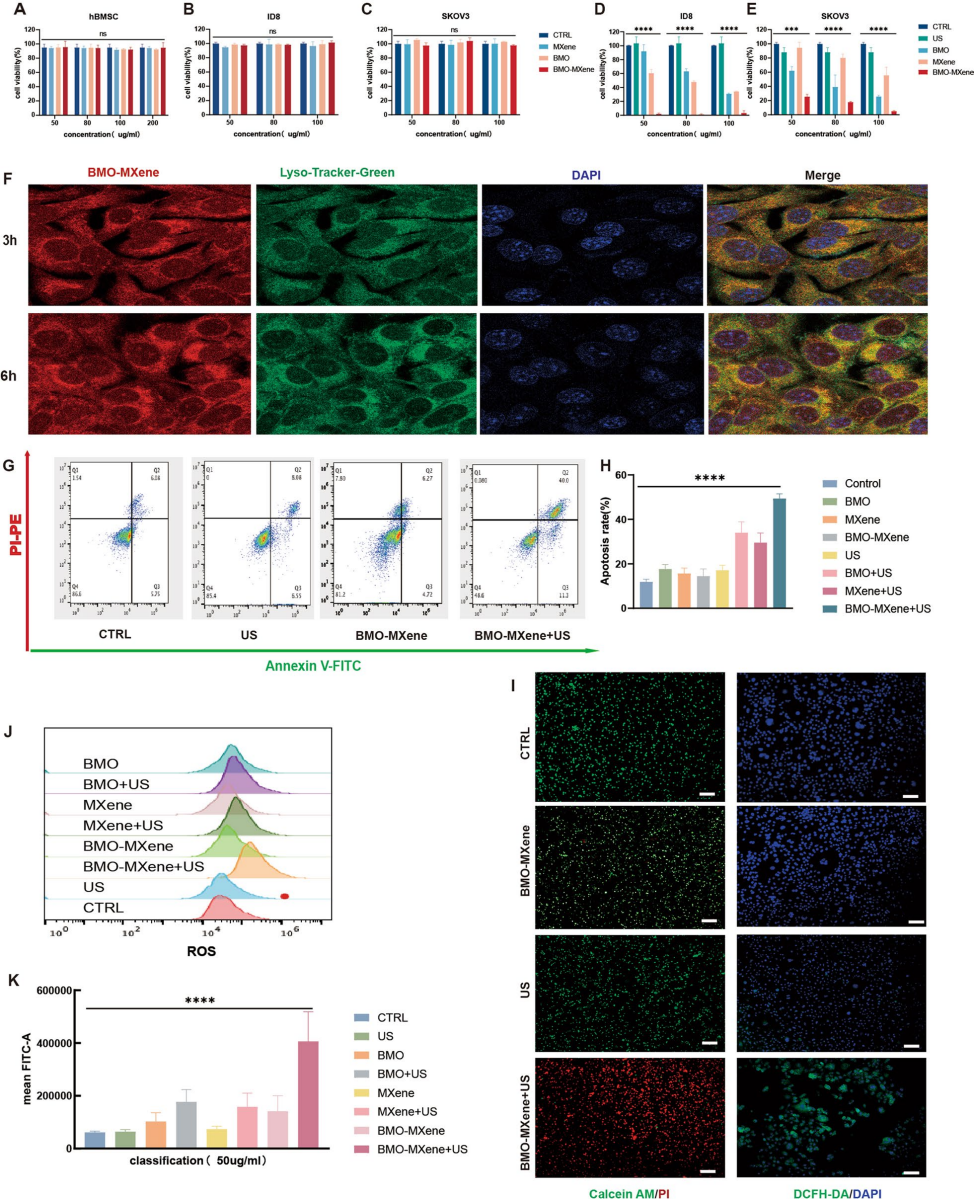
The antitumor performance of BMO-MXene in vitro. **A** Cytotoxicity of BMO, MXene and BMO-MXene (50 μg/mL, 80 μg/mL, 100 μg/mL, 200 μg/mL) on hBMSC. **B, C** Cell viability of ID8 and SKOV3 cells under different treatment including BMO, MXene and BMO-MXene (50 μg/mL, 80 μg/mL, 100 μg/mL) without us irradiation. **D, E** Cell viability of ID8 and SKOV3 cells under different treatment including BMO, MXene and BMO-MXene (50 μg/mL, 80 μg/mL, 100 μg/mL) with US irradiation (1.5 W/cm^2^, 1.0 MHz, 50% duty cycle, 3 min). **F** Fluorescence co-localization of BMO-MXene and lysosomes. **G** The apoptosis rate of different treatments tested by flow cytometry using fluorescein-annexin-V and PI staining kit. **H** Apoptosis rate chart. **I** Fluorescence images of ID8 cells stained with DCFH-DA and Calcein AM/PI after various treatments. Scar bar: 100 µm **J, K**: measurement of ROS level by flow cytometry and relevant quantitative ROS analysis (data are presented as means ± SEM, and analyzed by one-way ANOVA; *P < 0.05, **P < 0.01, ***P < 0.001, nsP > 0.05)

ROS are critical regulators of tumor cell proliferation, self-renewal, differentiation, apoptosis, and drug resistance [50]. High ROS levels can be detrimental to tumor cells, inducing cell death. Considering the excellent SDT performance of BMO-MXene, we investigated its anti-tumor effects against OC. The cytotoxicity of the nanosheets was assessed at different concentrations using Cell Counting Kit-8 (CCK8) assays. The viability of hBMSCs was not significantly affected by BMO-MXene, even at concentrations up to 200 µg/ml (Fig. 5A), indicating minimal side effects on healthy cells. The nanosheets did not significantly affect the viability of ID8 cells (Fig. 5B) or SKOV3 cells (Fig. 5C). In addition, US treatment (1.5 W/cm^2^, 1.0 MHz, 50% duty cycle, 3 min) alone did not inhibit tumor cell growth (Fig. 5D, E). However, when combined with nanosheets, particularly BMO-MXene, cell death induced by US stimulation was enhanced. At a BMO-MXene concentration of 50 μg/mL, the respective survival rates of ID8 cells and cisplatin-resistant SKOV3 OC cells were less than 10% and 30%, respectively. Notably, BMO-MXene exhibited higher antitumor efficiency than BMO or MXene alone. Moreover, BMO-MXene co-localization with lysosomes decreased between 3 and 6 h after the addition of the BMO-MXene complex. These results suggest that BMO-MXene entered cells and subsequently entered the cytoplasm, indicating lysosomal escape (Fig. 5F).

To further explore the antitumor effects of BMO-MXene, live/dead staining assays were performed (Figs. 5I, S5). Consistent with the CCK8 experiments, the number of dead red cells significantly increased under US excitation after co-culturing the tumor cells with BMO-MXene. However, neither treatment with US nor the nanosheets alone (BMO, MXene, and BMO-MXene) exhibited significant cytotoxicity. Flow cytometry was then performed to analyze apoptosis (Fig. 5G, H, and S6), revealing a significant increase in the percentage of cells in the late-stage of apoptosis under the synergistic effects of BMO-MXene and US treatment. In contrast, BMO, MXene, or US treatment alone displayed a lesser capacity to induce apoptosis in OC cells. In addition, under US stimulation, BMO and MXene promoted apoptosis to a lesser extent than BMO-MXene.

Given the excellent ROS production capacity of BMO-MXene, ROS production was investigated in OC cells after different treatments, using DCFH-DA. The BMO-MXene + US group exhibited the strongest green fluorescence intensities (Figs. 5I, S7), and flow cytometric assays further confirmed that BMO-MXene induced the highest ROS production under US excitation (Fig. 5J, K). These results demonstrated that the piezoelectric acoustic-sensitive NP, BMO-MXene, induced excessive ROS production in tumor cells under US excitation, thereby inhibiting tumor cell proliferation and promoting tumor cell death.

### In vivo antitumor performance

**Fig. 6:**
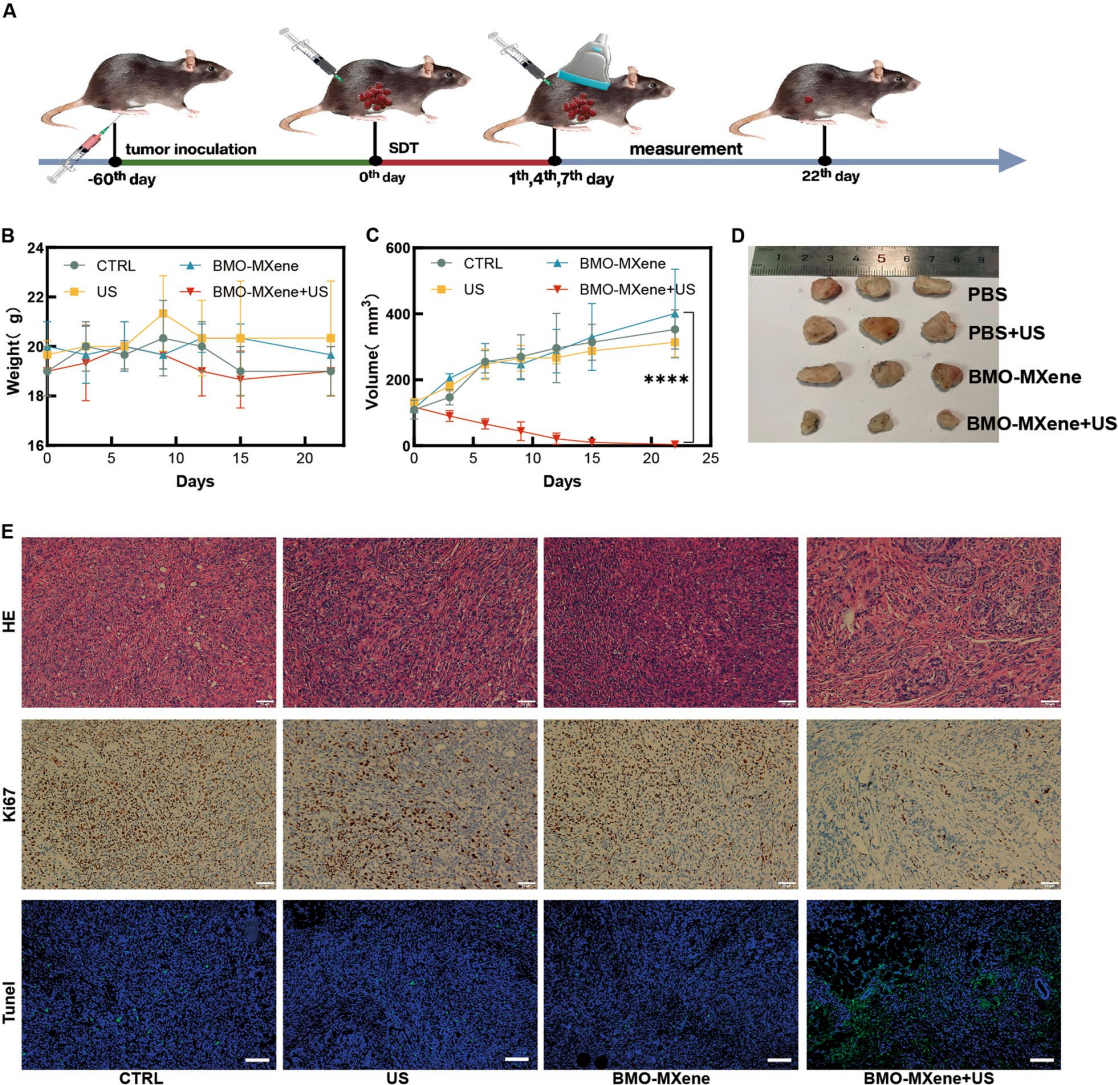
The antitumor performance of BMO-MXene in vivo.** A** Schematic diagram of animal experiments. **B** Body weight curve within 22 days. **C** Tumor growth curves within 22 days after different treatments. **D** Images of tumors in different treatment groups after 22 days. **E** Representative images of H&E staining, antigen Ki67 staining, and Tunel immunofluorescence staining of tumor sections in different experimental group. Scar bar: 50 µm (data are presented as means ± SEM, and analyzed by one-way ANOVA; *P < 0.05, **P < 0.01, ***P < 0.001, nsP > 0.05)

The antitumor effects of BMO-MXene were further evaluated in mice bearing ID8 tumor-cell xenografts. Subcutaneously transplanted tumors are frequently used in preclinical studies owing to their ease of observation, making them a standard model for evaluating therapeutic interventions. Given the extensive use of subcutaneous transplanted tumors in preclinical studies of OC to investigate the in vivo antitumor effects and associated mechanisms of various treatments [51–53], we evaluated the antitumor effects of the BMO-MXene by establishing a subcutaneous ID8 tumor model in female C57BL/6J mice. The tumor-bearing mice were randomly divided into four groups treated with (1) phosphate-buffered saline (PBS), (2) PBS + US irradiation for 15 min, (3) BMO-MXene, or (4) BMO-MXene + US irradiation for 15 min. Intratumoral injection was selected as the treatment modality to rapidly achieve high local drug (i.e., BMO-MXene) concentrations in tumor tissues after injection, prevent significant systemic exposure and off-target toxicity, enable full drug activity, and reduce drug damage to vital organs [54]. BMO-MXene was intratumorally injected (5 mg/kg) and subjected to US irradiation (1.5 W/cm^2^, 1.0 MHz, 50% duty cycle, 15 min) 24 h post-injection. This cycle was repeated three times, with a 3-day interval between each treatment (Fig. 6A). Body weights (Fig. 6B) and tumor volumes of the mice (Fig. 6C, D) were recorded every three days. We observed that changes in body weight after treatment were not significantly different from those in the control group. However, tumor volumes were significantly reduced after BMO-MXene treatment with US exposure compared to control treatment. These preliminary findings suggested that BMO-MXene exhibited excellent in vivo antitumor effects following US excitation. Moreover, Hematoxylin and eosin (H&E) and immunohistochemical staining for tumor pathology revealed that significantly lower Ki-67 expression and significantly higher TUNEL staining in the tumor tissues of the BMO-MXene + US group than in the other three groups. These results further support the notion that BMO-MXene could significantly promote tumor cell death under US excitation (Fig. 6E).

### Antitumor mechanism of BMO-MXene

**Fig. 7:**
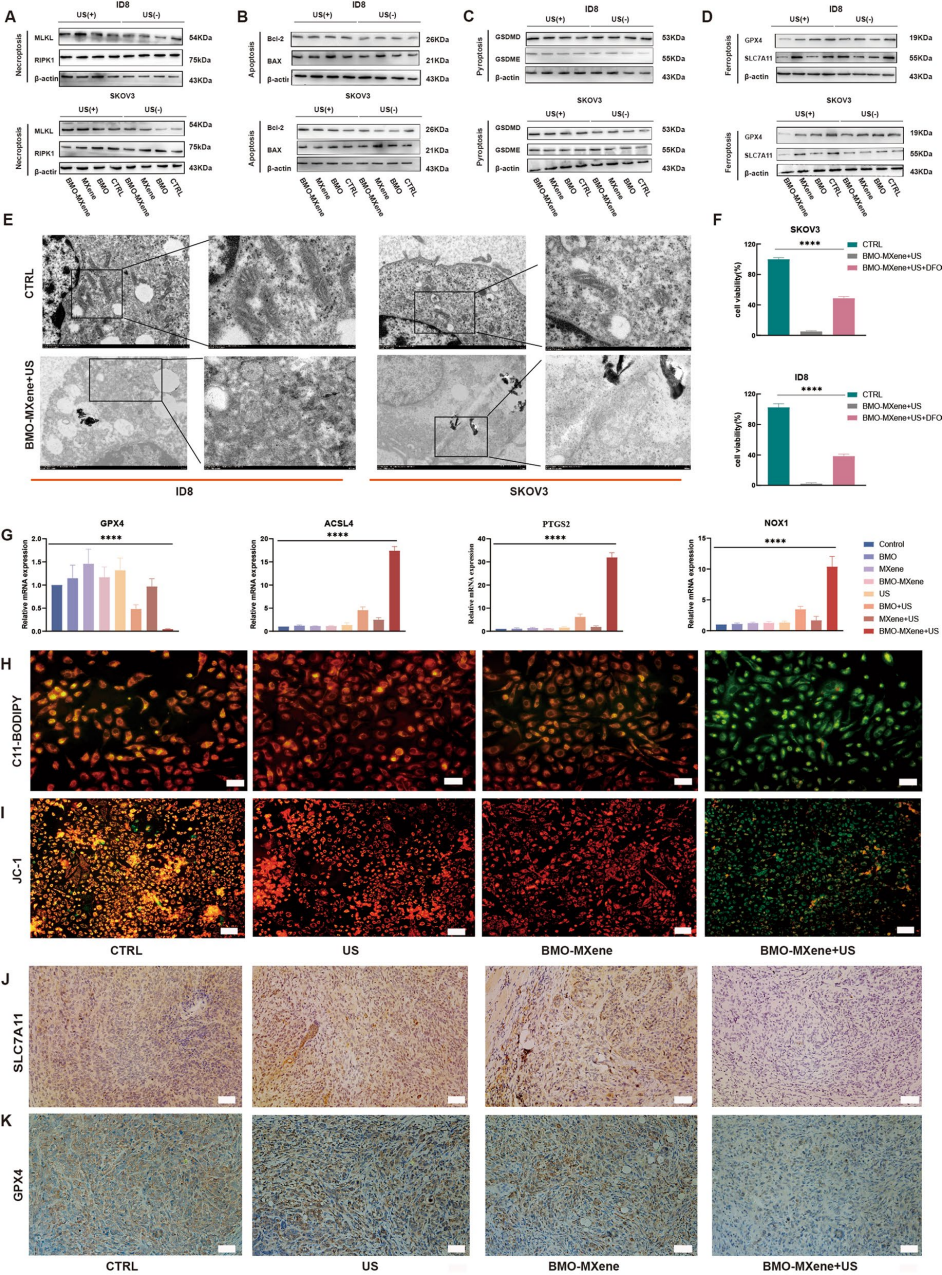
Antitumor mechanisms. **A–D** RIPK1, MLKL (**A**), BAX, Bcl-2 (**B**), GSDMD, GSDME (**C**), GPX4, SLC7A11 (**D**) expression levels of ID8 cells and SKOV3 cells measured by Western Blot. **E** The morphological changes of ID8 cells and SKOV3 cells observed by Transmission electron microscopy.** F** The viability of ID8 cells and SKOV3 cells assayed by CCK8 after BMO-MXene + US treatment in the presence or absence of DFO. **G** GPX4, ACSL4, PTGS2 and NOX1 gene expression levels detected by qRT-PCR after different treatments. **H** Lipid peroxidation levels of ID8 cells after different treatments detected by C11-BODIPY assays. Scar bar: 50 µm **I** Mitochondrial membrane potential of different treatments tested by JC-1 kits. Scar bar: 100 µm. **J-K** GPX4 and SLC7A11 expression levels of tumor issue assessed by immunohistochemical staining. Scar bar: 50 µm (data are presented as means ± SEM, and analyzed by one-way ANOVA; *P < 0.05, **P < 0.01, ***P < 0.001, nsP > 0.05)

BMO-MXene induced ROS accumulation in tumor cells under US excitation [55, 56]. This suggested that it may disrupt the intracellular redox balance and promote the metabolic reprogramming of tumor cells, inducing a shift from aerobic glycolysis to mitochondrial metabolism, thereby initiating ferroptosis [57]. Therefore, we investigated a series of biomarkers to ascertain the type of death the occurring in OC cells. Biomarkers of necroptosis (Fig. 7A), apoptosis (Fig. 7B), and pyroptosis (Fig. 7C) exhibited negligible trends in all treatment groups, whereas biomarkers of ferroptosis were significantly downregulated in the BMO-MXene + US group (Fig. 7D). Moreover, these results were further confirmed by IHC staining for glutathione peroxidase (GPX4) and cystathionine transporter protein (SLC7A11) (Fig. 7J, K).

Transmission electron microscopy was used to observe morphological changes in the mitochondria of OC cells after BMO-MXene + US treatment. The findings revealed a significant disappearance of mitochondrial cristae following disappeared significantly after BMO-MXene + US treatment (Fig. 7E). Moreover, deferrioxamine (DFO) was used to determine whether the biological functions of BMO-MXene + US could be reversed. Notably, the effects of combined BMO-MXene and US treatment, which promoted cell viability, were partially reversed by DFO (Fig. 7F).

In addition, total RNA was extracted from OC cells in each group, and the expression of molecules associated with ferroptosis signaling (GPX4, PTGS2, NOX1, and ACSL4) was verified via qRT-PCR. PTGS, NOX1, and ACSL4 were significantly upregulated by BMO-MXene + US treatment, whereas GPX4 was significantly downregulated (Fig. 7G). Ferroptosis is iron-dependent and induced by lipid peroxidation [58]. Therefore, lipid peroxidation levels are an important measure of ferroptosis. In our study, intracellular lipid peroxidation levels and antioxidant properties were evaluated using C11 BODIPY 581/591. The BMO-MXene + US group exhibited the strongest green fluorescence signal, indicating that US combined with BMO-MXene treatment upregulated lipid peroxidation levels most effectively in tumor cells (Fig. 7H, S8, S9). Ferroptosis is often accompanied by decreased mitochondrial membrane potential, and JC-1 staining suggested that the BMO-MXene group exhibited the strongest green fluorescence signal in the cytoplasm under US excitation. This result suggested that the BMO-MXene group exhibited the strongest ability to induce a decrease in the mitochondrial membrane potential in OC cells (Fig. 7I, S10, S11). These data further suggest that BMO-MXene + US treatment can cause ferroptosis in OC cells.

During ferroptosis, GSH depletion decreases GPX4 activity and leads to impaired cellular antioxidant capacity, resulting in metabolic dysfunction due to excess LPO and ROS levels. These unique characteristics of the cell-death mechanism distinguish it from several other forms of programmed cell death and enable ferroptosis to circumvent apoptosis/necroptosis resistance [9]. Importantly, cancer cells undergoing ferroptosis usually release damage-associated molecular patterns (DAMPs), which favors ICD-induced cell death and promotes dendritic cell (DC) maturation and enhanced CD8^+^ T cell invasion into tumors [59].

**Fig. 8:**
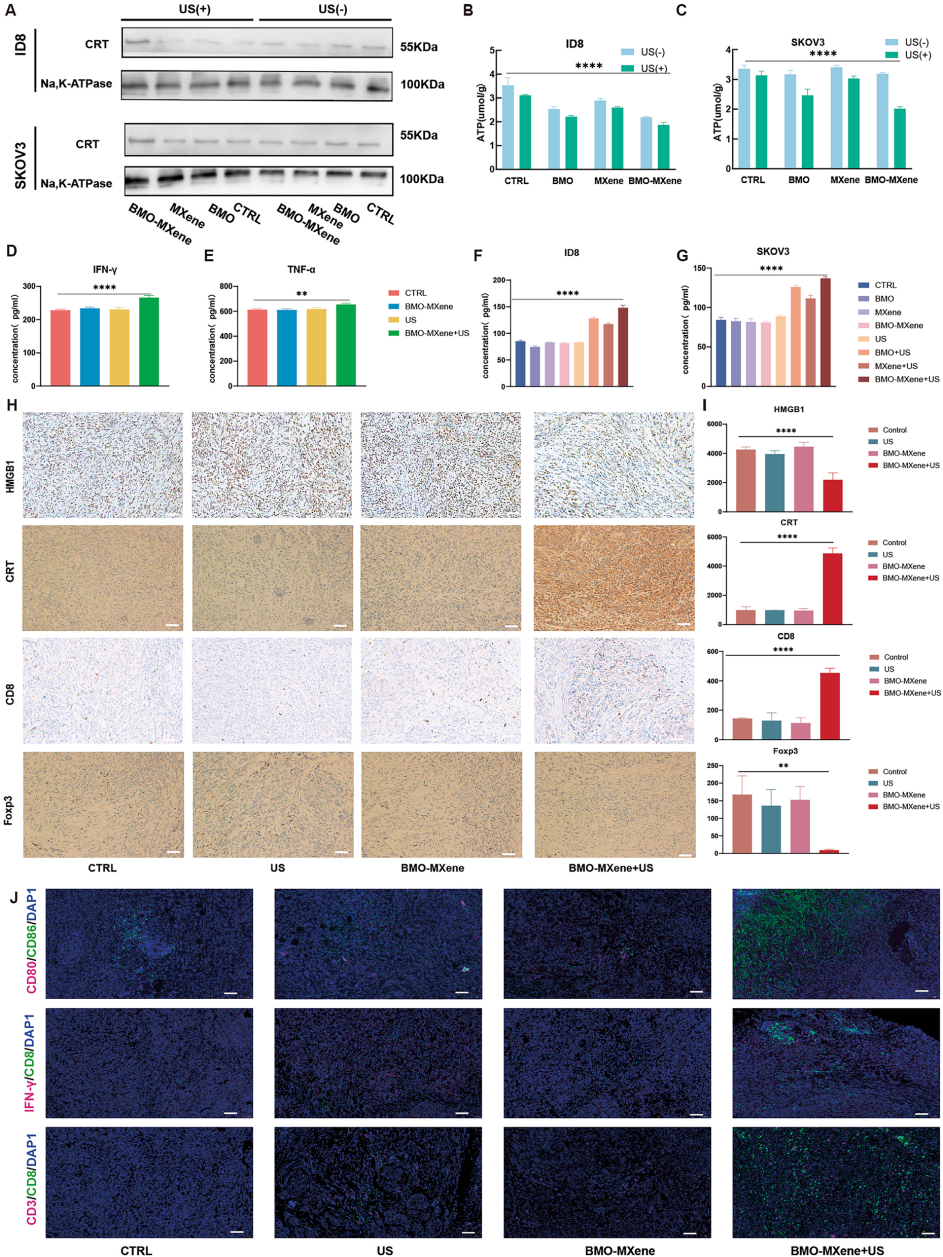
Antitumor mechanisms. **A** CRT expression level of ID8 cells and SKOV3 cells measured by Western Blot. **B, C** Intracellular ATP level of ID8 and SKOV3 cells with different treatments. **D, E** Serum levels of IFN-γ and TNF-α in mice from different treatment groups. **F, G** ELISA kit for detection the levels of HMGB1 secreted by ID8 and SKOV3 cells with different treatments. **H** Immunohistochemistry staining showing the expression level of HMGB1, CRT, Foxp3, and CD8 on ovarian tumor issue. **I** Expression level chart of Foxp3, CRT, CD8, and HMGB1 on ovarian tumor issue. **J** Immunofluorescence images of CD3^+^CD8^+^T cells, CD80^+^CD86^+^DC cells, IFN-γ^+^CD8^+^T cells in tumor tissue slices after different treatments. Scar bar: 50 µm (data are presented as means ± SEM, and analyzed by one-way ANOVA; *P < 0.05, **P < 0.01, ***P < 0.001, nsP > 0.05)

Therefore, the effectiveness of the BMO-MXene + US strategy for ICDs was further investigated by measuring three key ICD signals in OC cells, namely cell-membrane surface calreticulin (CRT) exposure, high mobility group box-1 protein (HMGB1) release, and adenosine triphosphate (ATP) secretion. CRT expression on the surface of OC cell membranes was higher in the BMO-MXene + US group than in the control group (Fig. 8A). Moreover, ATP assay kits were used to measure intracellular ATP contents, revealing that the BMO-MXene + US group had the lowest intracellular ATP contents, suggesting that the BMO-MXene + US strategy effectively promoted ATP secretion (Fig. 8B, C). In addition, ELISA results indicated that BMO-MXene + US treatment induced the highest degree of HMGB1 release (Fig. 8F, G). IHC staining for CRT and HMGB1 in tumor tissues confirmed that BMO-MXene + US treatment stimulated ICD in tumor cells (Fig. 8H, I).

ICD, necrosis, and pyroptosis can significantly promote antitumor immunity [60]. The proportion of mature DCs (CD80^+^CD86^+^) in the tumor microenvironment significantly increased after BMO-MXene + US administration, suggesting the recruitment of immune cells to activate an adaptive immune response. DCs are antigen-presenting cells that initiate T cell functions [61], and both IHC and IF staining revealed significant recruitment of CD8^+^ T cells (CD3^+^CD8^+^, IFN-γ^+^CD8^+^) in the BMO-MXene + US treatment group [62], along with significantly fewer Treg cells (Fig. 8H–J). The expression levels of the inflammatory factors TNF-α and IFN-γ were also measured using ELISA, and the results, confirming that BMO-MXene + US treatment increased TNF-α and IFN-γ secretion (Fig. 8D, E).

In conclusion, immune responses induced by SDT, ferroptosis, and ICD mainly involved the promotion of DC maturation, followed by increased cytotoxic T lymphocyte infiltration and decreased suppressor T cells in tumor tissues.

### Biocompatibility of BMO-MXene

**Fig. 9:**
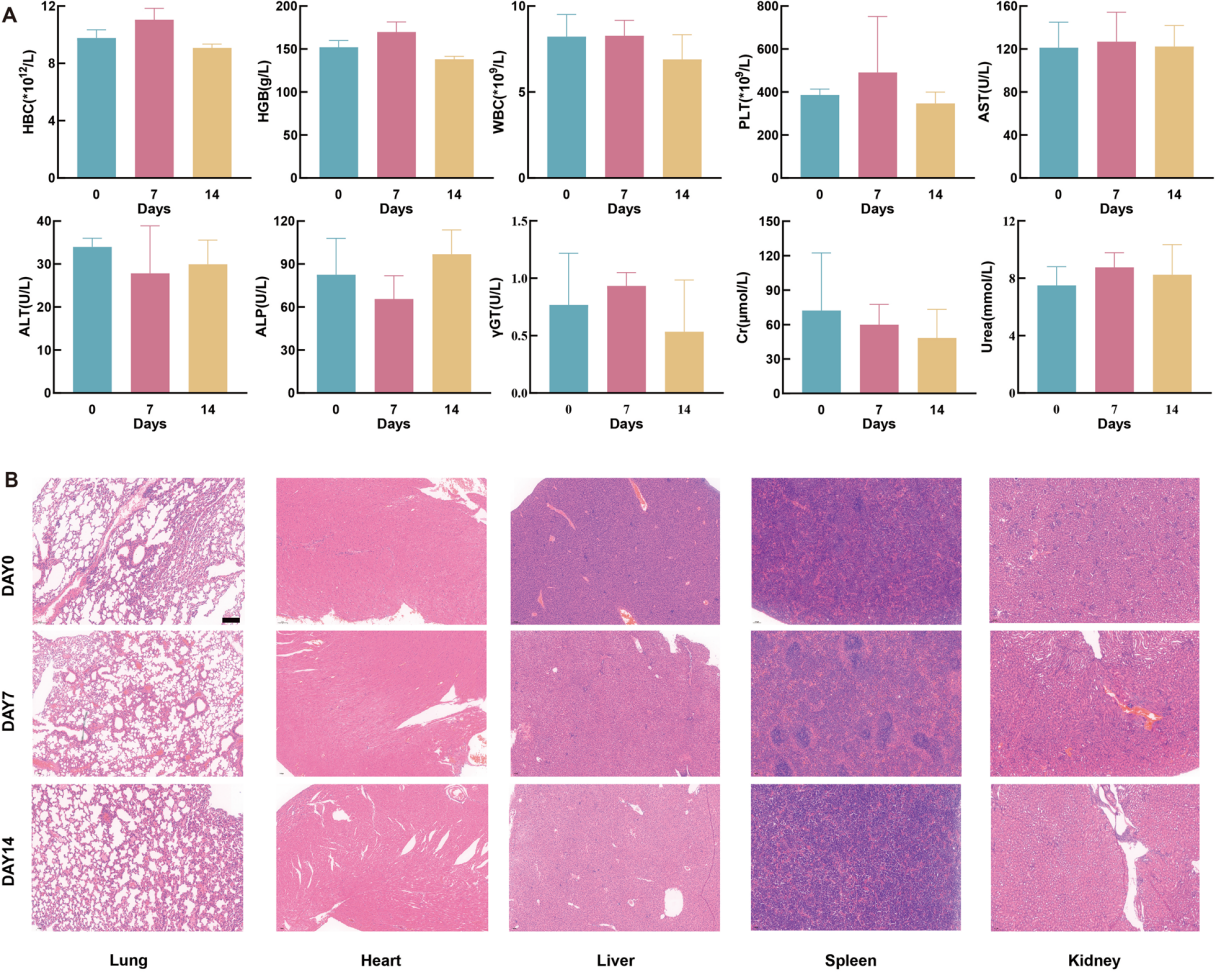
Biocompatibility of BMO-MXene. **A** Analysis of blood routine, liver function and kidney function in mice at day 0, day 7, and day 14. **B** H&E staining analysis of essential organs on day 0, day 7, and day 14. Scar bar: 100 µm

The effects of BMO-MXene on vital organs in mice must be carefully considered. Therefore, we initially assessed its systemic toxic effects by comparing changes in blood cell parameters, blood biochemistry indices, and H&E staining of the major mouse organs before and after BMO-MXene injection. The liver and kidney function indices and blood parameters of the mice remained within normal ranges before and after BMO-MXene treatment (Fig. 9A). These preliminary findings indicated that BMO-MXene treatment exhibited little effect on liver and kidney function and hematology of mice. In addition, the vital organs (heart, spleen, liver, kidneys, and lungs) of mice were isolated at different treatment times and subjected to H&E staining to observe the effect of BMO-MXene + US on the vital organs of mice. As shown in Fig. 9B, BMO-MXene+US treatment imposed a minimal burden on the vital organs. Therefore, BMO-MXene exhibits a favorable safety profile as a novel antitumor therapeutic agent, laying the foundation for its subsequent clinical translation.

## Conclusion

In summary, this study developed a 2D sonosensitizer (BMO-MXene) with dual activation efficacy for ferroptosis and ICD. This technique enables the precise targeting of tumor cells upon tumor localization and application of exogenous US. The Schottky heterojunction formed between BMO and MXene improved the efficiency of electron–hole separation and inhibited electron–hole complexation, resulting in a high ROS yields and potent antitumor properties. Acoustic-catalyzed generation of ROS disturbed the redox homeostasis of OC cells and downregulated GPX4 and SLC7A11 expression levels. Under US excitation, BMO-MXene induced the release of DAMPs and inflammatory factors, activated ICD, and improved the tumor immune microenvironment. Our strategy provides a noninvasive, precise, and novel tool for OC treatment based on SDT. In the clinical settings, the BMO-MXene nanosystem could precisely and efficiently induce OC cell death with the assistance of US. We are confident that a more comprehensive understanding of the interactions between BMO-MXene and organisms as well as tumor tissues based on this study can help promote breakthroughs of BMO-MXene in SDT of solid tumors.

### Supplementary Information

Supplementary Material 1.

## Data Availability

The data that support the findings of this study are available from the corresponding author upon reasonable request.

## References

[1] Webb PM, Jordan SJ (2024). Global epidemiology of epithelial ovarian cancer. Nat Rev Clin Oncol.

[2] Doherty JA, Peres LC, Wang C, Way GP, Greene CS, Schildkraut JM (2017). Challenges and opportunities in studying the epidemiology of ovarian cancer subtypes. Curr Epidemiol Rep.

[3] Kuroki L, Guntupalli SR (2020). Treatment of epithelial ovarian cancer. BMJ.

[4] Li H, Xiao Y, Li Q, Yao J, Yuan X, Zhang Y, Yin X, Saito Y, Fan H, Li P (2022). The allergy mediator histamine confers resistance to immunotherapy in cancer patients via activation of the macrophage histamine receptor H1. Cancer Cell.

[5] Borgers JSW, Heimovaara JH, Cardonick E, Dierickx D, Lambertini M, Haanen JBAG, Amant F (2021). Immunotherapy for cancer treatment during pregnancy. Lancet Oncol.

[6] Kuroki L, Guntupalli SR (2020). Treatment of epithelial ovarian cancer. BMJ (Clin Res Ed).

[7] Disis ML, Taylor MH, Kelly K, Beck JT, Gordon M, Moore KM, Patel MR, Chaves J, Park H, Mita AC (2019). Efficacy and safety of avelumab for patients with recurrent or refractory ovarian cancer: phase 1b results from the JAVELIN solid tumor trial. JAMA Oncol.

[8] Barber E, Matei D (2021). Immunotherapy in ovarian cancer: we are not there yet. Lancet Oncol.

[9] Jiang X, Stockwell BR, Conrad M (2021). Ferroptosis: mechanisms, biology and role in disease. Nat Rev Mol Cell Biol.

[10] Tang D, Chen X, Kang R, Kroemer G (2021). Ferroptosis: molecular mechanisms and health implications. Cell Res.

[11] Hong T, Lei G, Chen X, Li H, Zhang X, Wu N, Zhao Y, Zhang Y, Wang J (2021). PARP inhibition promotes ferroptosis via repressing SLC7A11 and synergizes with ferroptosis inducers in BRCA-proficient ovarian cancer. Redox Biol.

[12] Zhang C, Liu X, Jin S, Chen Y, Guo R (2022). Ferroptosis in cancer therapy: a novel approach to reversing drug resistance. Mol Cancer.

[13] Wang C-K, Chen T-J, Tan GYT, Chang F-P, Sridharan S, Yu C-HA, Chang Y-H, Chen Y-J, Cheng L-T, Hwang-Verslues WW (2023). MEX3A mediates p53 degradation to suppress ferroptosis and facilitate ovarian cancer tumorigenesis. Cancer Res.

[14] Wiernicki B, Maschalidi S, Pinney J, Adjemian S, Vanden Berghe T, Ravichandran KS, Vandenabeele P (2022). Cancer cells dying from ferroptosis impede dendritic cell-mediated anti-tumor immunity. Nat Commun.

[15] Zhou Y, Chen K, Lin WK, Liu J, Kang W, Zhang Y, Yang R, Jin L, Cheng Y, Xu A, Wang W (2023). Photo-enhanced synergistic induction of ferroptosis for anti-cancer immunotherapy. Adv Healthcare Mater.

[16] Efimova I, Catanzaro E, Van der Meeren L, Turubanova VD, Hammad H, Mishchenko TA, Vedunova MV, Fimognari C, Bachert C, Coppieters F (2020). Vaccination with early ferroptotic cancer cells induces efficient antitumor immunity. J Immunothera Cancer.

[17] Liu J, Zhan J, Zhang Y, Huang L, Yang J, Feng J, Ding L, Shen Z, Chen X (2023). Ultrathin clay nanoparticles-mediated mutual reinforcement of ferroptosis and cancer immunotherapy. Adv Mater (Deerfield Beach, Fla).

[18] Niu X, Chen L, Li Y, Hu Z, He F (2022). Ferroptosis, necroptosis, and pyroptosis in the tumor microenvironment: perspectives for immunotherapy of SCLC. Semin Cancer Biol.

[19] Van Coillie S, Van San E, Goetschalckx I, Wiernicki B, Mukhopadhyay B, Tonnus W, Choi SM, Roelandt R, Dumitrascu C, Lamberts L (2022). Targeting ferroptosis protects against experimental (multi)organ dysfunction and death. Nat Commun.

[20] Xie S, Sun W, Zhang C, Dong B, Yang J, Hou M, Xiong L, Cai B, Liu X, Xue W (2021). Metabolic control by heat stress determining cell fate to ferroptosis for effective cancer therapy. ACS Nano.

[21] Hassannia B, Vandenabeele P, Vanden Berghe T (2019). Targeting ferroptosis to iron out cancer. Cancer Cell.

[22] Li H, Wang B, Wu S, Dong S, Jiang G, Huang Y, Tong X, Yu M (2023). Ferroptosis is involved in polymyxin B-induced acute kidney injury via activation of p53. Chem Biol Interact.

[23] Zhou L, Xue X, Hou Q, Dai C (2022). Targeting ferroptosis attenuates interstitial inflammation and kidney fibrosis. Kidney Diseases (Basel, Switzerland).

[24] Zhen X, Cheng P, Pu K (2019). Recent advances in cell membrane-camouflaged nanoparticles for cancer phototherapy. Small.

[25] Vaughan HJ, Green JJ, Tzeng SY (2020). Cancer-targeting nanoparticles for combinatorial nucleic acid delivery. Adv Mater (Deerfield Beach, Fla).

[26] Xiao Y, Yu D (2021). Tumor microenvironment as a therapeutic target in cancer. Pharmacol Ther.

[27] Garcia Garcia CJ, Huang Y, Fuentes NR, Turner MC, Monberg ME, Lin D, Nguyen ND, Fujimoto TN, Zhao J, Lee JJ (2022). Stromal HIF2 regulates immune suppression in the pancreatic cancer microenvironment. Gastroenterology.

[28] Boedtkjer E, Pedersen SF (2020). The acidic tumor microenvironment as a driver of cancer. Annu Rev Physiol.

[29] Zhou F, Feng B, Yu H, Wang D, Wang T, Ma Y, Wang S, Li Y (2019). Tumor microenvironment-activatable prodrug vesicles for nanoenabled cancer chemoimmunotherapy combining immunogenic cell death induction and CD47 blockade. Adv Mater (Deerfield Beach, Fla).

[30] Sun C-C, Zhu W, Li S-J, Hu W, Zhang J, Zhuo Y, Zhang H, Wang J, Zhang Y, Huang S-X (2020). FOXC1-mediated LINC00301 facilitates tumor progression and triggers an immune-suppressing microenvironment in non-small cell lung cancer by regulating the HIF1α pathway. Genome Med.

[31] Harris AL (2002). Hypoxia—a key regulatory factor in tumour growth. Nat Rev Cancer.

[32] Sun D, Zhou S, Gao W (2020). What went wrong with anticancer nanomedicine design and how to make it right. ACS Nano.

[33] Shi J, Kantoff PW, Wooster R, Farokhzad OC (2017). Cancer nanomedicine: progress, challenges and opportunities. Nat Rev Cancer.

[34] Liang S, Yao J, Liu D, Rao L, Chen X, Wang Z (2023). Harnessing nanomaterials for cancer sonodynamic immunotherapy. Adv Mater (Deerfield Beach, Fla).

[35] Yang Y, Huang J, Liu M, Qiu Y, Chen Q, Zhao T, Xiao Z, Yang Y, Jiang Y, Huang Q, Ai K (2023). Emerging sonodynamic therapy-based nanomedicines for cancer immunotherapy. Adv Sci (Weinheim, Baden-Wurttemberg, Germany).

[36] Wu T, Liu Y, Cao Y, Liu Z (2022). Engineering macrophage exosome disguised biodegradable nanoplatform for enhanced sonodynamic therapy of glioblastoma. Adv Mater (Deerfield Beach, Fla).

[37] Ji C, Si J, Xu Y, Zhang W, Yang Y, He X, Xu H, Mou X, Ren H, Guo H (2021). Mitochondria-targeted and ultrasound-responsive nanoparticles for oxygen and nitric oxide codelivery to reverse immunosuppression and enhance sonodynamic therapy for immune activation. Theranostics.

[38] Zeng Z, Zhang C, He S, Li J, Pu K (2022). Activatable cancer sono-immunotherapy using semiconducting polymer nanobodies. Adv Mater (Deerfield Beach, Fla).

[39] Ning S, Dai X, Tang W, Guo Q, Lyu M, Zhu D, Zhang W, Qian H, Yao X, Wang X (2022). Cancer cell membrane-coated C-TiO_2_ hollow nanoshells for combined sonodynamic and hypoxia-activated chemotherapy. Acta Biomater.

[40] Lu J, Xu C, Li F, Yang Z, Peng Y, Li X, Que M, Pan C, Wang ZL (2018). Piezoelectric effect tuning on ZnO microwire whispering-gallery mode lasing. ACS Nano.

[41] Dong Y, Dong S, Liu B, Yu C, Liu J, Yang D, Yang P, Lin J (2021). 2D Piezoelectric Bi(2) MoO(6) nanoribbons for GSH-enhanced sonodynamic therapy. Adv Mater.

[42] Ma W, Yao BH, Zhang W, He YQ, Yu Y, Niu JF (2021). Fabrication of PVDF-based piezocatalytic active membrane with enhanced oxytetracycline degradation efficiency through embedding few-layer E-MoS2 nanosheets. Chem Eng J.

[43] Shi Z, Ge Y, Yun Q, Zhang H (2022). Two-dimensional nanomaterial-templated composites. Acc Chem Res.

[44] Riazi H, Taghizadeh G, Soroush M (2021). MXene-based nanocomposite sensors. ACS Omega.

[45] Ran J, Zhang J, Yu J, Jaroniec M, Qiao SZ (2014). Earth-abundant cocatalysts for semiconductor-based photocatalytic water splitting. Chem Soc Rev.

[46] Hantanasirisakul K, Gogotsi Y (2018). Electronic and optical properties of 2D transition metal carbides and nitrides (MXenes). Adv Mater (Deerfield Beach, Fla).

[47] Ran J, Gao G, Li F-T, Ma T-Y, Du A, Qiao S-Z (2017). Ti3C2 MXene co-catalyst on metal sulfide photo-absorbers for enhanced visible-light photocatalytic hydrogen production. Nat Commun.

[48] Zhang Z, Yates JT (2012). Band bending in semiconductors: chemical and physical consequences at surfaces and interfaces. Chem Rev.

[49] Guo J, Shi L, Zhao J, Wang Y, Tang K, Zhang W, Xie C, Yuan X (2018). Enhanced visible-light photocatalytic activity of Bi2MoO6 nanoplates with heterogeneous Bi2MoO6-x@Bi2MoO6 core-shell structure. Appl Catal B.

[50] Cheung EC, Vousden KH (2022). The role of ROS in tumour development and progression. Nat Rev Cancer.

[51] Cao Q, Wang W, Zhou M, Huang Q, Wen X, Zhao J, Shi S, Geng K, Li F, Hatakeyama H (2020). Induction of antitumor immunity in mice by the combination of nanoparticle-based photothermolysis and anti-PD-1 checkpoint inhibition. Nanomed Nanotechnol Biol Med.

[52] Brentville VA, Metheringham RL, Daniels I, Atabani S, Symonds P, Cook KW, Vankemmelbeke M, Choudhury R, Vaghela P, Gijon M (2020). Combination vaccine based on citrullinated vimentin and enolase peptides induces potent CD4-mediated anti-tumor responses. J Immunothera Cancer.

[53] Kim DY, Kwon DY, Kwon JS, Park JH, Park SH, Oh HJ, Kim JH, Min BH, Park K, Kim MS (2016). Synergistic anti-tumor activity through combinational intratumoral injection of an in-situ injectable drug depot. Biomaterials.

[54] Zhu P, Chen Y, Shi J (2020). Piezocatalytic tumor therapy by ultrasound-triggered and BaTiO-mediated piezoelectricity. Adv Mater (Deerfield Beach, Fla).

[55] Niu B, Liao K, Zhou Y, Wen T, Quan G, Pan X, Wu C (2021). Application of glutathione depletion in cancer therapy: enhanced ROS-based therapy, ferroptosis, and chemotherapy. Biomaterials.

[56] Wang Y, Qi H, Liu Y, Duan C, Liu X, Xia T, Chen D, Piao H-L, Liu H-X (2021). The double-edged roles of ROS in cancer prevention and therapy. Theranostics.

[57] Liu J, Kuang F, Kroemer G, Klionsky DJ, Kang R, Tang D (2020). Autophagy-dependent ferroptosis: machinery and regulation. Cell Chem Biol.

[58] Li D, Li Y (2020). The interaction between ferroptosis and lipid metabolism in cancer. Signal Transduct Target Ther.

[59] Tang R, Xu J, Zhang B, Liu J, Liang C, Hua J, Meng Q, Yu X, Shi S (2020). Ferroptosis, necroptosis, and pyroptosis in anticancer immunity. J Hematol Oncol.

[60] Xiong H, Ma X, Wang X, Su W, Wu L, Zhang T, Xu Z, Sun Z-J (2021). Inspired epigenetic modulation synergy with adenosine inhibition elicits pyroptosis and potentiates. Cancer Immunothera.

[61] Li X, Khorsandi S, Wang Y, Santelli J, Huntoon K, Nguyen N, Yang M, Lee D, Lu Y, Gao R (2022). Cancer immunotherapy based on image-guided STING activation by nucleotide nanocomplex-decorated ultrasound microbubbles. Nat Nanotechnol.

[62] St Paul M, Ohashi PS (2020). The roles of CD8+ T cell subsets in antitumor immunity. Trends Cell Biol.

