# Supplementary material for: Ultrasound-responsive Bi2MoO6-MXene heterojunction as ferroptosis inducers for stimulating immunogenic cell death against ovarian cancer
Source: J Nanobiotechnology. 2024 Jul 11;22:408. doi: 10.1186/s12951-024-02658-3 (PMC11238442; doi:10.1186/s12951-024-02658-3)
Supplement: Supplementary file 1 — Supplementary Material 1. [file 12951_2024_2658_MOESM1_ESM.docx]

**Supporting information**

**Ultrasound-responsive Bi_2_MoO_6_-MXene heterojunction as ferroptosis inducers for stimulating immunogenic cell death against ovarian cancer**

*Shuangshuang Cheng^1#^, Ting Zhou^1#^, Yue Luo^3#^, Jun Zhang^1^, Kejun Dong^1^, Qi Zhang^1^, Wan Shu^1^, Tangansu Zhang^1^, Qian Zhang^1^, Rui Shi^1^, Yuwei Yao^1^, Hongbo Wang^1,2^.*

1. Department of Obstetrics and Gynecology, Union Hospital, Tongji Medical College, Huazhong University of Science and Technology, Wuhan 430022, China
2. Clinical Research Center of Cancer Immunotherapy, Hubei, Wuhan, 430022, China. E-mail : [drwanghb69@hust.edu.cn](mailto:drwanghb69@hust.edu.cn)
3. Biomedical Materials Engineering Research Center, Collaborative Innovation Center for Advanced Organic Chemical Materials Co-constructed by the Province and Ministry, Hubei Key Laboratory of Polymer Materials, Ministry-of-Education Key Laboratory for the Green Preparation and Application of Functional Materials, School of Materials Science & Engineering, Hubei University, Wuhan 430062, China


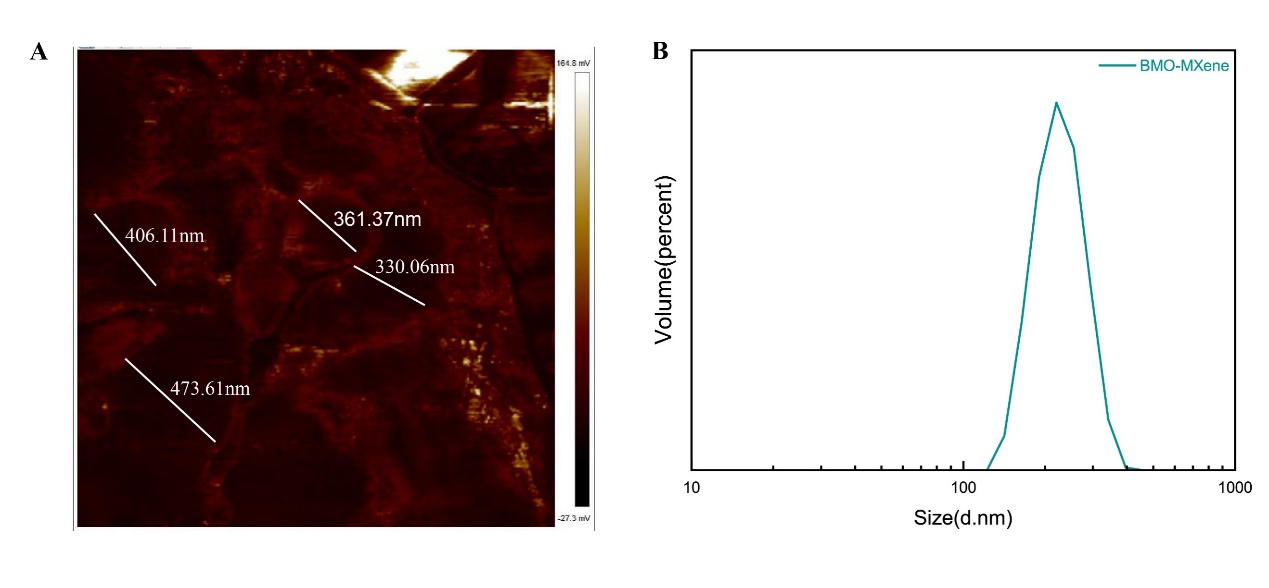


**FigureS1:** **A** nanosize of BMO-MXene based on AFM; **B** DLS of BMO-MXene.

**
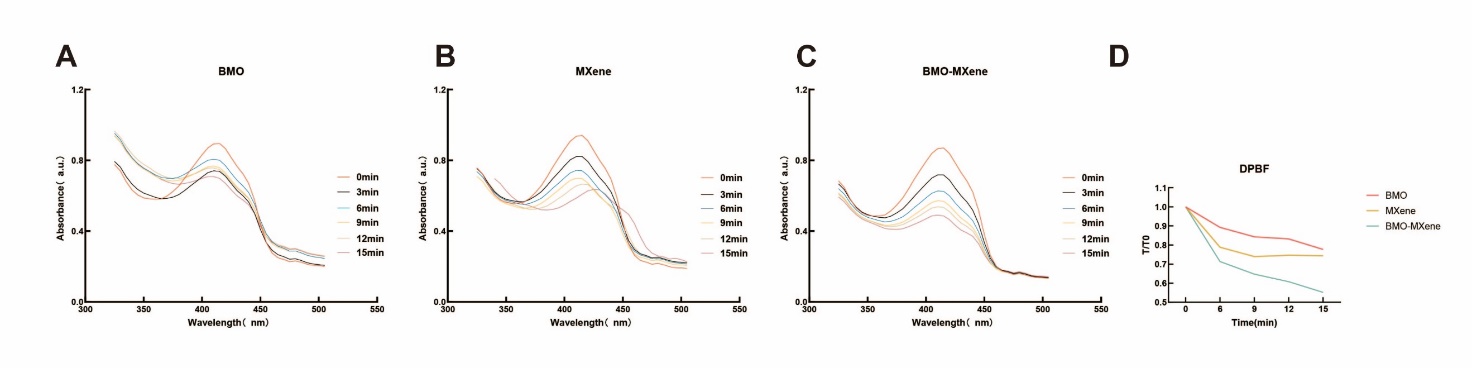
**

**FigureS2:** The ability of nanosheetss to produce singlet oxygen detected by DPBF degradation under US irradiation (**A-C**). **D** Rate of DPBF degradation by BMO, MXene and BMO-MXene under US excitation.

**
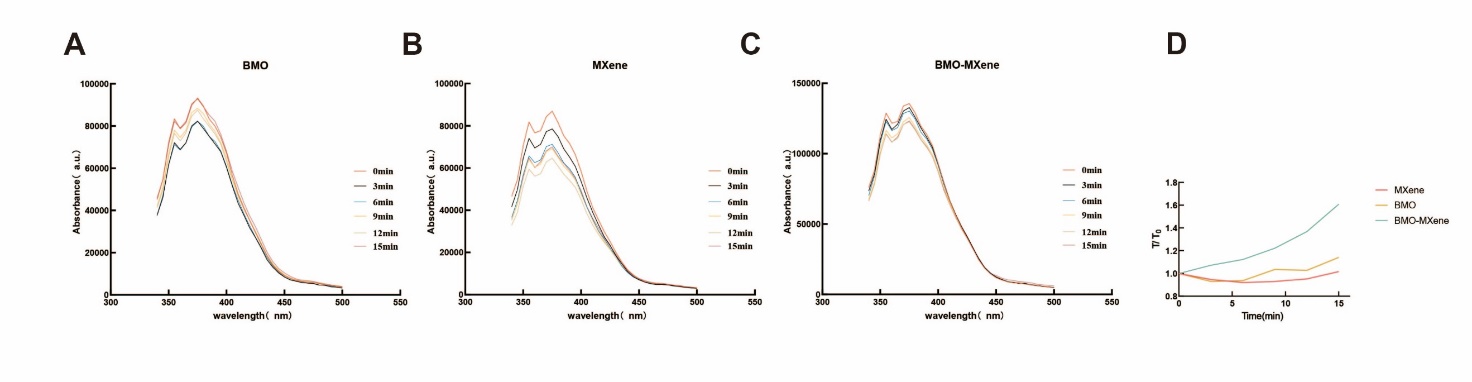
**

**FigureS3:** The ability of nanoparticles to produce hydroxyl radicals detected by TA under US irradiation (**A-C**). **D** Rate of TA by BMO, MXene and BMO-MXene under US excitation.

**
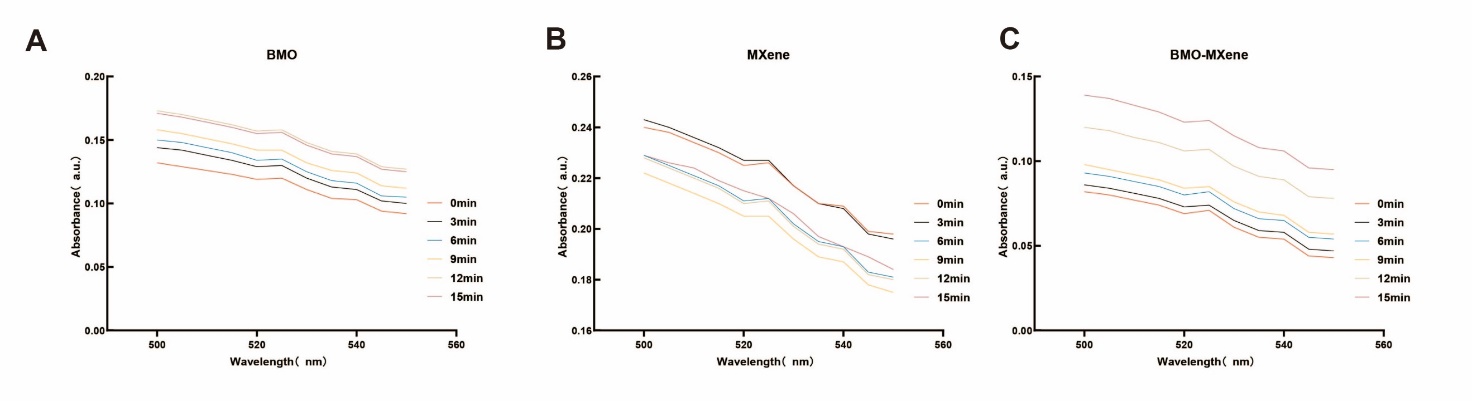
**

**FigureS4:** The ability of nanoparticles to produce hydroxyl radicals detected by NBT under US irradiation (**A-C**).

**
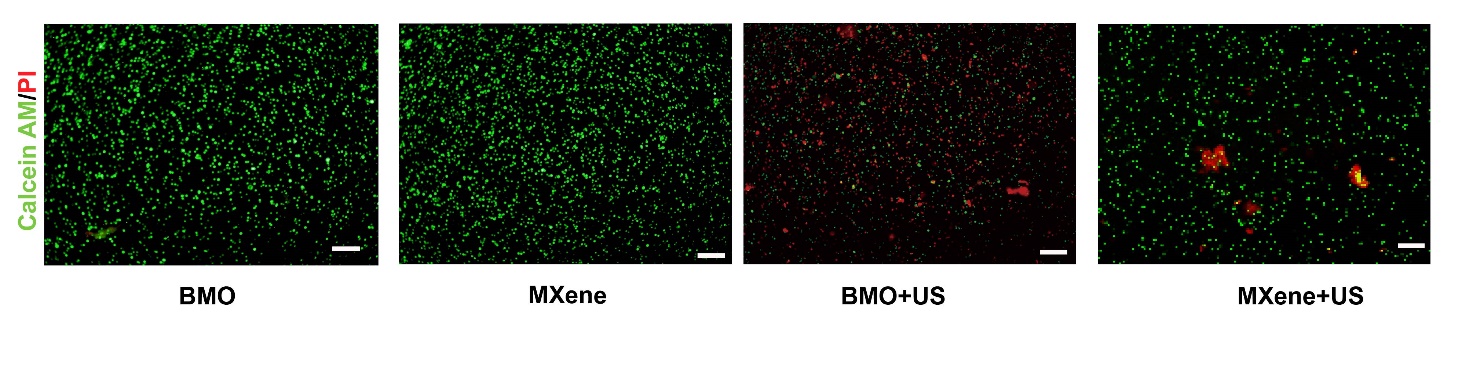
**

**FigureS5:** Fluorescence images of ID8 cells stained with Calcein AM/PI after various treatments. (Scar bar: 100um)


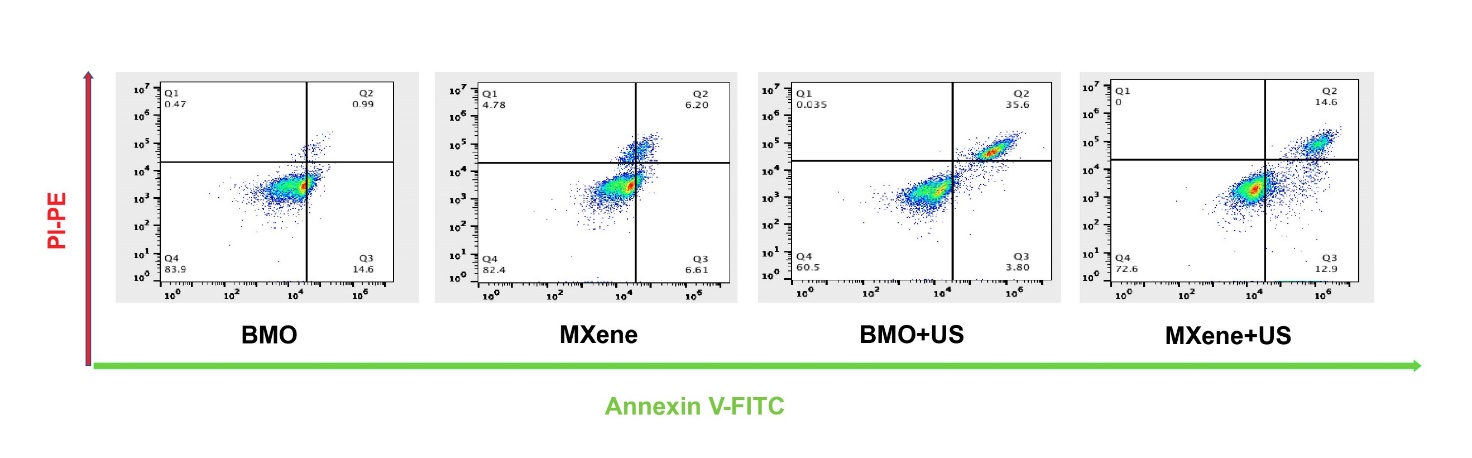


**FigureS6:** The apoptosis rate of different treatments tested by flow cytometry using fluorescein-annexin-V and PI staining kit.

**
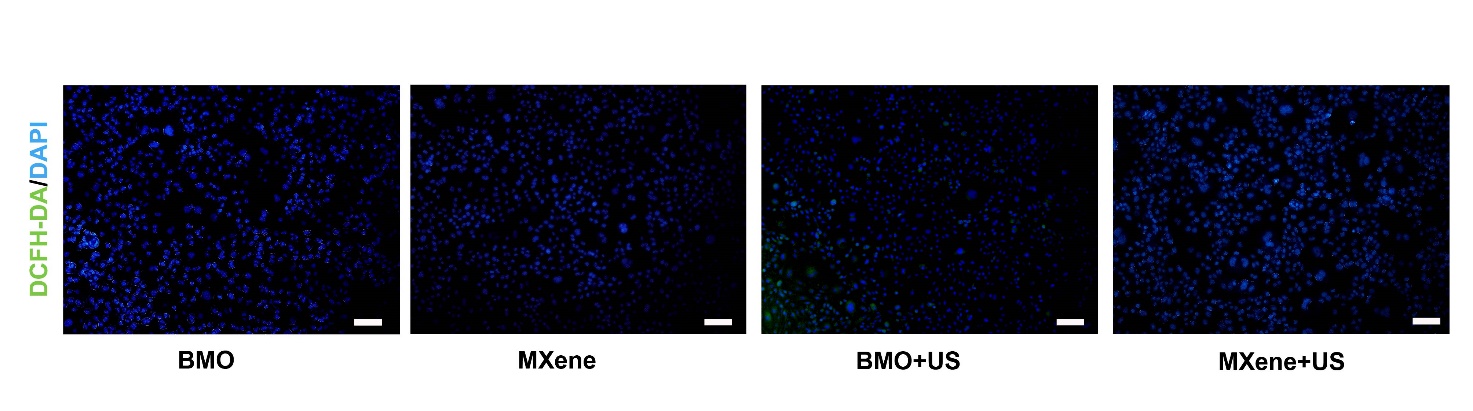
**

**FigureS7:** Fluorescence images of ID8 cells stained with DCFH-DA after various treatments. (Scar bar: 100um)


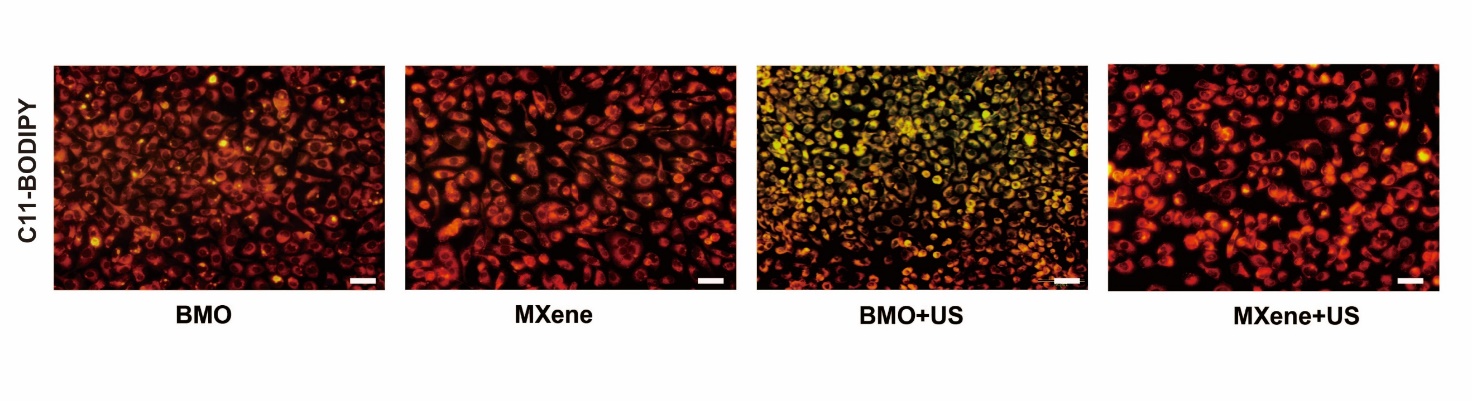


**FigureS8:** Lipid peroxidation levels of ID8 cells after different treatments detected by C11-BODIPY assays. (Scar bar: 50um)

**
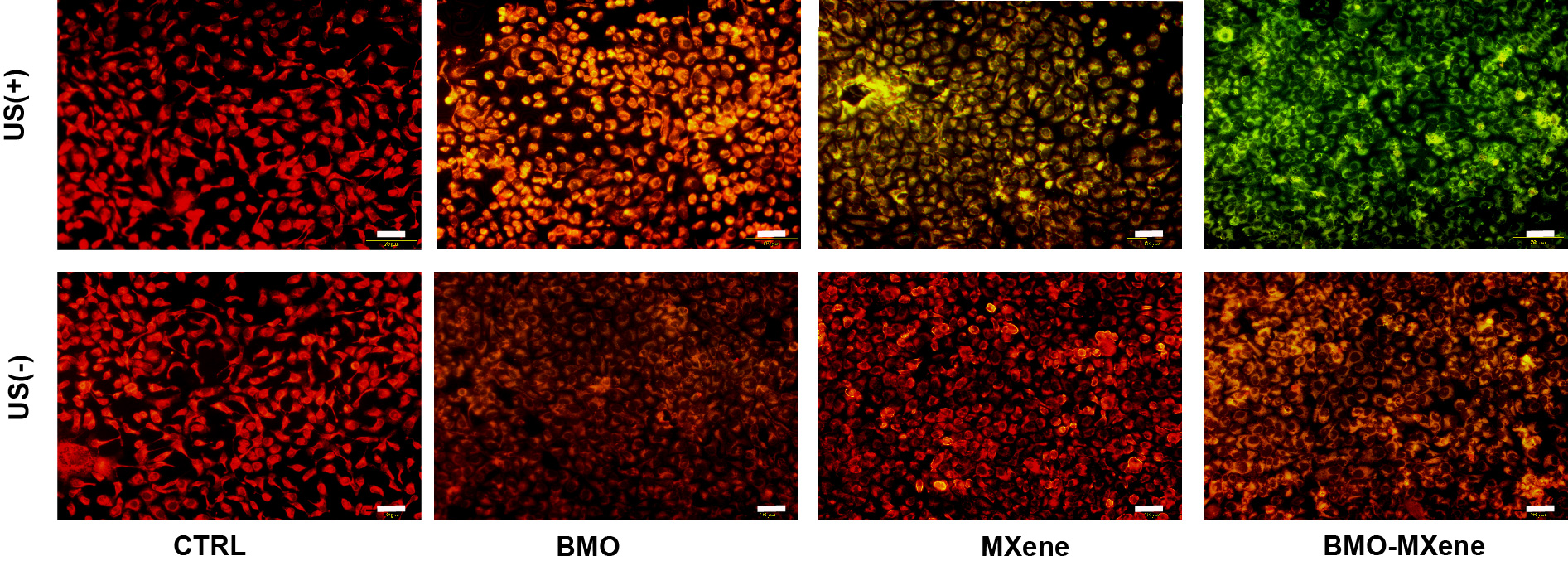
**

**FigureS9:** Lipid peroxidation levels of SKOV3 cells after different treatments detected by C11-BODIPY assays. (Scar bar: 100um)


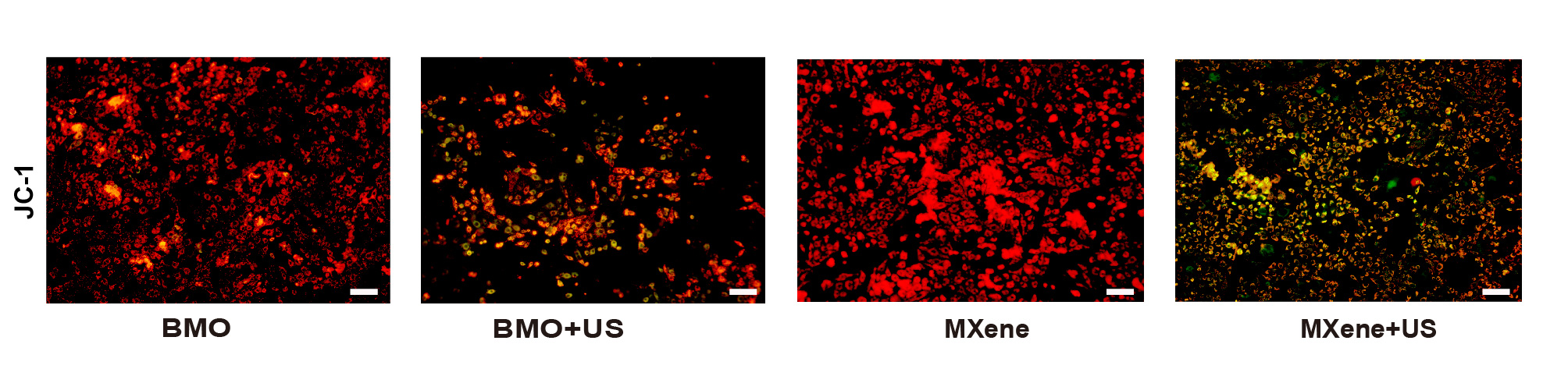


**FigureS10:** Mitochondrial membrane potential of different treatments tested by JC-1 kits in ID8 cells. (Scar bar: 100um)


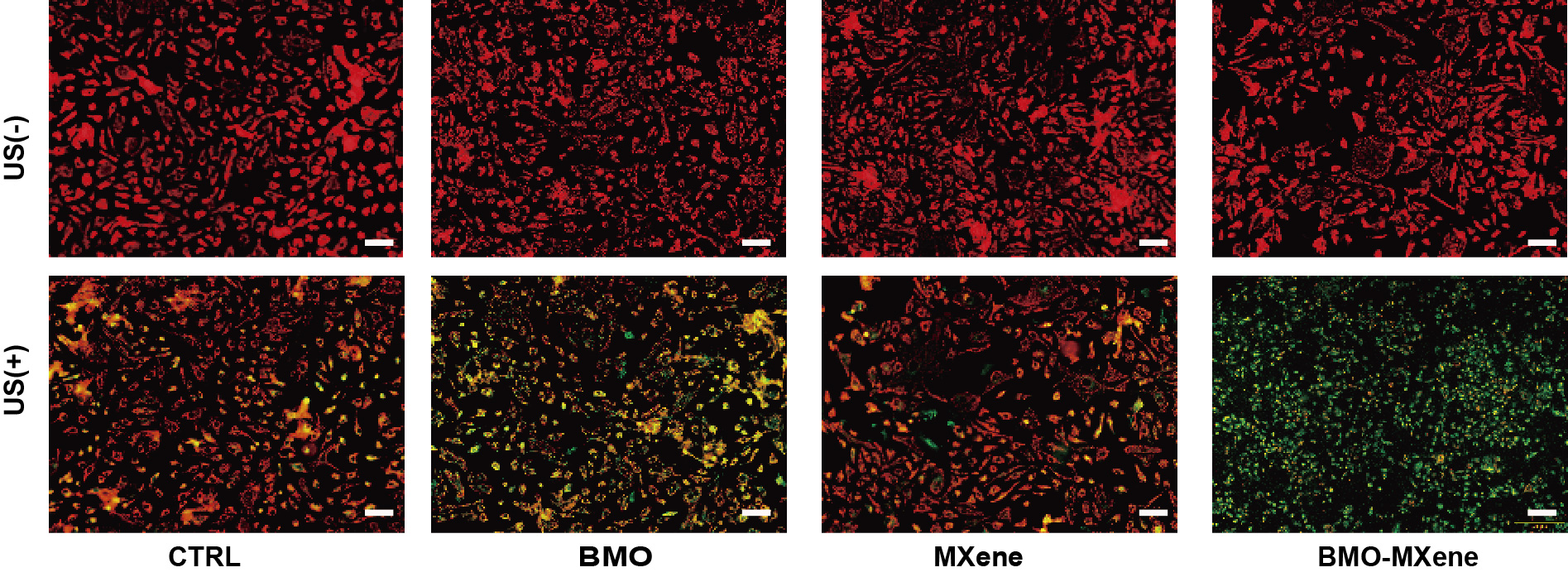


**FigureS11:** Mitochondrial membrane potential of different treatments tested by JC-1 kits in SKOV3 cells. (Scar bar: 100um)
